# In Silico Integrated Systems Biology Analysis of Gut-Derived Metabolites from Philippine Medicinal Plants Against Atopic Dermatitis

**DOI:** 10.3390/ijms262110731

**Published:** 2025-11-04

**Authors:** Legie Mae Soriano, Kumju Youn, Mira Jun

**Affiliations:** 1 Department of Health Sciences, The Graduate School of Dong-A University, Busan 49315, Republic of Korea; 2473012@donga.ac.kr; 2 Department of Food Science and Nutrition, Dong-A University, Busan 49315, Republic of Korea; kjyoun@dau.ac.kr; 3 Center for Food & Bio Innovation, Dong-A University, Busan 49315, Republic of Korea

**Keywords:** atopic dermatitis, gut-skin axis, network pharmacology, molecular docking, molecular dynamics, density functional theory

## Abstract

Atopic dermatitis (AD) is a multifactorial skin disorder characterized by immune and barrier dysfunction. The gut–skin axis is a bidirectional pathway through which gut and skin influence each other via microbial metabolites. Bioactive metabolites produced by microbial transformation of phytochemicals show potential for AD prevention. This study developed a computational systems biology pipeline that prioritized gut-derived metabolites from Philippine medicinal plants by integrating metabolite prediction, pharmacokinetics, network analysis, and molecular simulations. From 2231 predicted metabolites, 31 satisfied pharmacological criteria and were mapped to 199 AD-associated targets, with ALB, CASP3, and PPARG identified as hub genes. Two metabolites, THPOC and PM38, exhibited complementary target affinities and strong binding stability. THPOC stabilized ALB and CASP3, supporting barrier integrity and apoptosis regulation, while PM38 strongly engaged PPARG, modulating lipid metabolism and anti-inflammatory transcription. They exhibited comparable or superior docking scores, stable MD interactions, and favorable binding free energies, compared to abrocitinib, an approved AD treatment. DFT analysis confirmed electronic stability and donor–acceptor properties linked to target selectivity. These findings highlight THPOC and PM38 as promising immunometabolic modulators acting on key AD-related pathways. Collectively, this study introduces a reproducible systems-based computational discovery framework, offering a novel preventive strategy for AD.

## 1. Introduction

Atopic dermatitis (AD) is a chronic, relapsing inflammatory skin disorder characterized by recurrent eczematous lesions and intense pruritus [1]. Globally, AD affects approximately 15–20% of children and up to 10% of adults, highlighting its substantial public health burden [2,3]. Although AD is not fatal, its chronic symptoms, such as persistent itching, sleep disturbances, social embarrassment, and depression, significantly affect patients’ psychological wellbeing and social relationships [4]. Its pathogenesis involves a complex interplay of epidermal barrier disruption, immune dysregulation, genetic susceptibility, and environmental factors. The bidirectional interaction between immune activation and epidermal barrier impairment has emerged as a critical driver of disease progression. In particular, the predominance of type 2 helper T cell (Th2)-mediated immune responses represents a hallmark feature of AD, driving chronic inflammation through the overproduction of cytokines [e.g., interleukin (IL)-4, IL-5, and IL-13] and contributing to immunological changes such as eosinophilia and elevated serum IgE levels [5].

Expanding on the Th2-mediated immune dysregulation, emerging evidence highlights the gut–skin axis as a bidirectional communication linking the gut microbiota and systemic immune function. Through this axis, microbial metabolites including short chain fatty acids (SCFAs), tryptophan catabolites, and bile acid derivatives have been shown to regulate cytokine production, promote regulatory T cell differentiation, and maintain epithelial junction integrity [6]. These metabolites act as signaling intermediaries that modulate both systemic inflammation and cutaneous immune responses. Disruptions in gut microbiota and its metabolites have been associated with increased epithelial permeability, heightened Th2-type inflammation, and colonization by skin pathogens in AD [7]. These findings suggest that targeting gut microbiota and its metabolites could provide novel ways to modulate immune responses in AD.

Traditional AD treatments include topical corticosteroids, calcineurin inhibitors, and immunosuppressants such as cyclosporine and methotrexate. While these agents are effective in reducing symptoms, a “one-size-fits-all” approach to disease care remains prevalent, and they are associated with side effects including skin atrophy, systemic immunosuppression, nephrotoxicity, and increased susceptibility to infections. The chronic nature of AD, along with heterogeneous treatment responses and limited therapeutic options, complicates disease management [8]. Moreover, conventional animal models do not adequately reflect the transcriptomic and signaling pathways observed in human AD skin, and fundamental structural and immunological differences between mouse and human skin further limit translational relevance [9]. These limitations underscore the need for alternative approaches such as systems biology and computational modeling to investigate disease mechanisms in clinically relevant contexts [10]. Such approaches enable the systematic exploration of molecular networks underlying disease, facilitating the identification of clinically relevant targets and predictive biomarkers.

Plant-derived bioactive compounds are increasingly recognized as promising candidates for therapeutic development due to their structural diversity, favorable safety profiles, cost-effectiveness, and multi-target mechanisms of action. Notably, phytochemicals such as epigallocatechin gallate (EGCG), curcumin, and glycyrrhizin have demonstrated anti-inflammatory and barrier-restoring effects in eczema models [11]. While these compounds may exert direct effects, growing evidence suggests that some plant-derived compounds are converted into bioactive metabolites, which may exhibit distinct or enhanced immunomodulatory properties [12,13,14]. For instance, baicalein, a hydrolyzed form of baicalin, has been shown to reduce epidermal hyperplasia and immune cell infiltration in AD-like lesions while modulating cytokine production and promoting keratinocyte differentiation. These findings support the preventive potential of gut microbiota-derived metabolites of phytochemicals in AD.

In the Philippines, the Department of Health (DOH) has officially recommended ten medicinal plants for therapeutic use, including *Senna alata*, *Momordica charantia*, *Allium sativum*, *Psidium guajava*, *Vitex negundo*, *Combretum indicum*, *Blumea balsamifera*, *Ehretia microphylla*, *Peperomia pellucida*, and *Mentha cordifolia*. Among these, some species are represented by regionally recognized cultivars. *Momordica charantia* L., for instance, includes varieties such as Galaxy, Sta. Monica, Sta. Rita Strain L., and Trident 357, commonly grown in Luzon and Central Visayas [15]. *Allium sativum* L. is known locally through cultivars like Ilocos White, Batanes White, Mindoro White, and Nueva Ecija Pink, named after their respective provinces [16]. *Psidium guajava* L. is broadly categorized into red and white guava types cultivated nationwide, while *Vitex negundo* L. encompasses regional variants such as *V. trifolia*, *V. bicolor*, and *V. arvensis*, prevalent in Northern and Central Luzon [17,18]. For other species such as *Senna alata*, *Combretum indicum*, *Blumea balsamifera*, *Ehretia microphylla*, *Peperomia pellucida*, and *Mentha cordifolia*, no officially named local cultivars have been reported to date. These plants have demonstrated diverse activities such as anti-inflammatory, analgesic, metabolic, and antimicrobial effects that support their traditional use in treating skin infections, diabetes, hyperlipidemia, and gastrointestinal disorders [19]. Despite these promising findings, the pharmacological relevance of these compounds after gut microbial conversion remains largely unexplored.

Recent advances in integrative computational approaches have enabled a shift from single-compound, single-target paradigms to more multi-target frameworks for investigating how bioactive compounds modulate complex molecular networks [20]. This systems-level perspective is particularly valuable for studying DOH-recommended medicinal plants, which contain structurally diverse phytochemicals with potentially synergistic biological activities. Given the emerging role of gut-derived metabolites in modulating systemic immunity and skin inflammation, there is a compelling rationale to explore how metabolites from DOH-recommended medicinal plants may influence AD through the gut-skin axis. Rather than focusing solely on compounds with well-established individual activities, this study employs a systems pharmacology approach that emphasizes emergent, multi-target interactions across AD-relevant networks. This strategy reflects the foundational goal of network pharmacology, which is not to reaffirm known pharmacological effects but to uncover novel mechanisms that arise from compound–target–pathway interactions. While the pharmacological activities of individual DOH-recommended plants are recognized, this study emphasizes their collective effects following gut microbial transformation, reflecting the systems-level focus of network pharmacology.

**Figure 1:**
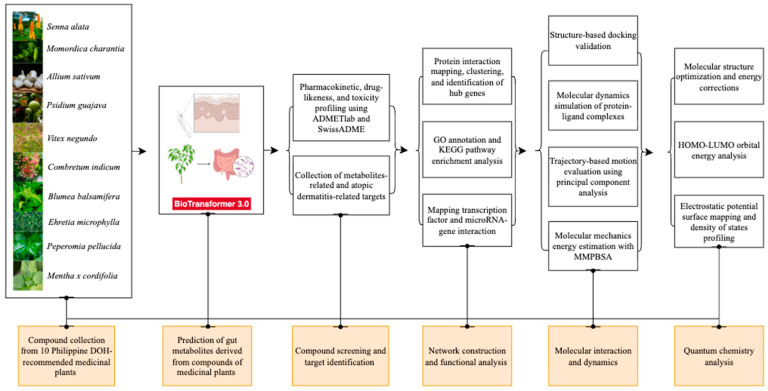
Overview of the comprehensive workflow.

The present study establishes a novel integrative in silico workflow to systematically characterize the AD preventive potential of gut microbiota metabolites from DOH-recommended medicinal plants. Candidate compounds were first prioritized based on predicted gut microbial conversion and absorption, distribution, metabolism, excretion, and toxicity (ADMET) profiles. Network pharmacology and gene regulatory mapping were employed to associate these metabolites with AD-related targets. Molecular docking provided initial structural validation by estimating binding affinity and orientation with key protein targets. Molecular dynamics (MD) simulations were subsequently performed to assess the temporal stability and interaction dynamics of the docked complexes under physiological conditions. In addition, density functional theory (DFT) analysis was used to characterize the electronic properties of the lead metabolites, offering quantum-level insight into their chemical reactivity and binding selectivity. This is the first study to model the microbial conversion of phytochemicals derived from DOH-recommended plants and their interaction with AD-relevant networks, providing novel insights into multi-target preventive strategies. The computational workflow is illustrated in Figure 1.

## 2. Results

### 2.1. Identification of Gut Microbiota-Derived Metabolites from 10 Confirmed DOH-Recommended Medicinal Plants and Associated Target Genes

A total of 824 compounds were retrieved from 10 medicinal plants recommended by the DOH of the Philippines, encompassing multiple structural classes, with flavonoids (5.3%), alkaloids (3.5%), phenolic compounds (12.0%), fatty acids (21.9%), and terpenoids (46.5%) constituting the largest proportions (Supplementary Table S1). To expand this chemically diverse dataset, in silico gut microbial metabolism prediction via BioTransformer v3.0, resulting in 2231 predicted metabolites (Table S2). After applying rigorous filtering based on physicochemical properties, drug-likeness criteria, and predicted toxicity profiles (Tables S3 and S4), a final set of 31 gut microbiota-derived metabolites was retained for target prediction and network analysis (Table 1).

**Figure 2:**
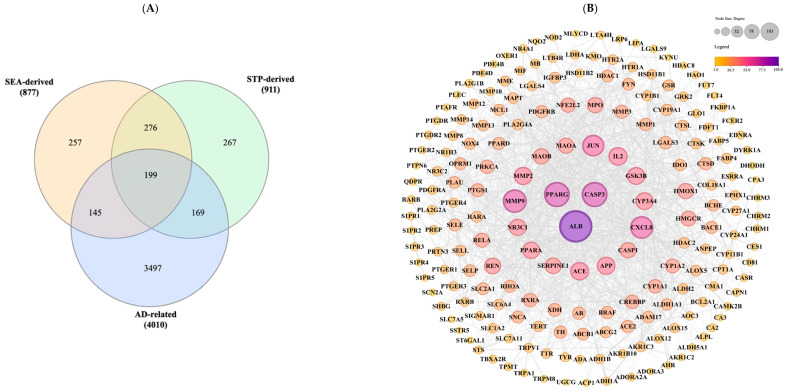
Network pharmacology analysis of overlapping gene targets. (**A**) Venn diagram illustrating the intersection of predicted targets from Similarity Ensemble Approach (SEA) and SwissTargetPrediction (STP) databases with genes associated with atopic dermatitis (AD); (**B**) Protein–protein interaction (PPI) network constructed using the STRING database (confidence score ≥ 0.4) and visualized in Cytoscape (v3.10.3). Node size and color intensity are proportional to degree centrality, with larger and darker-colored nodes representing higher connectivity and smaller, lighter-colored nodes indicating lower connectivity.

Potential protein targets of the 31 gut microbiota-derived metabolites were predicted using the SwissTargetPrediction (STP) and Similarity ensemble approach (SEA) (*p* < 8.5 × 10^−7^) platforms. This search yielded 877 targets from SEA and 911 from STP, with 475 unique protein targets retained after the removal of duplicates. For disease-specific target identification, the DisGeNET database (gene–disease association score ≥ 0.4) yielded 221 genes associated with AD, while the GeneCards database (relevance score ≥ 0.2) returned 4010 related genes. After removing redundant entries, a final set of 199 overlapping genes was obtained, representing shared targets between gut-derived metabolites and AD-associated genes. These common targets are illustrated in the Venn diagram (Figure 2A) and listed in Table S5.

### 2.2. Protein–Protein Interaction (PPI) Network Construction and Hub Gene Identification of Overlapping Gut Metabolite and AD-Associated Target Genes

The 199 overlapping targets between gut metabolite–associated proteins and AD-related genes were mapped into the STRING database (minimum confidence score ≥ 0.4) to construct PPI network (Figure 2B). The final network consisted of 193 nodes and 1719 edges, with six proteins excluded due to insufficient interaction confidence or disconnection from the network. The average node degree was 17.4, and the average local clustering coefficient was 0.451. The number of observed edges was substantially higher than the expected 606 edges, with a PPI enrichment *p*-value < 1 × 10^−16^, indicating statistically significant network connectivity.

Hub genes were prioritized separately for each of the five centrality metrics (degree, betweenness, closeness, stress, radiality) calculated in CytoHubba [23]. For each metric, the top 10 genes were extracted, and the intersection across all metrics was taken to identify consistently central nodes. This process yielded 10 hub genes (*ALB*, *CASP3*, *PPARG*, *MMP9*, *CXCL8*, *JUN*, *IL2*, *ACE*, *APP*, *MMP2*), which represent the most influential nodes in the AD-associated PPI network. Among them, *ALB*, *CASP3*, and *PPARG* ranked highest across multiple metrics and were selected as core candidates for subsequent validation analyses. Detailed topological scores are provided in Table 2, and the hub gene subnetwork is visualized in Figure S1.

### 2.3. Identification of Functional Modules and Enrichment Analysis

To determine whether hub genes were concentrated in specific functional modules, the PPI network was clustered using the Molecular Complex Detection (MCODE) algorithm in Cytoscape. Ten modules were identified, and the three most interconnected (Clusters 1–3) were selected for enrichment analysis based on their high MCODE scores and dense connectivity. Complete lists of significantly enriched Gene ontology (GO) terms and Kyoto encyclopedia of genes and genomes (KEGG) pathway are provided in Table S6.

**Figure 3:**
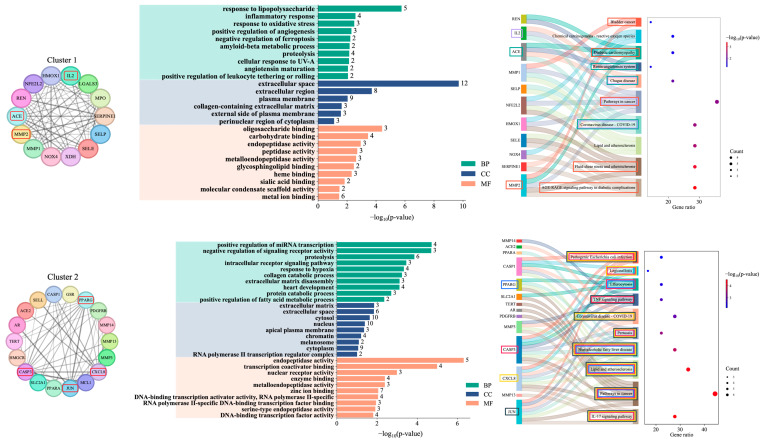

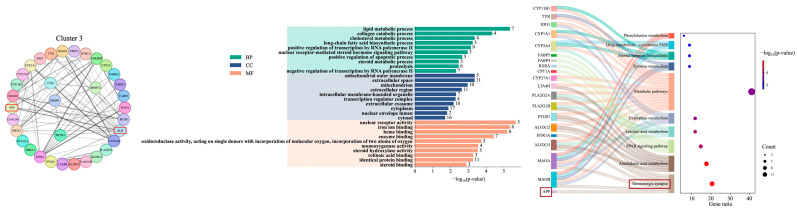
Functional modules identified from MCODE clustering of the PPI network and their enrichment analysis. For each cluster (Clusters 1–3), the left panel shows the PPI subnetwork (hub genes highlighted with red boxes), the middle panel presents GO enrichment results (BP: green, CC: blue, MF: peach), and the right panel shows KEGG pathway enrichment. Dot size reflects gene count; color intensity indicates −log_10_(*p*−value).

Cluster 1 contained *IL2*, *ACE*, and *MMP2*, positioned at highly connected nodes that mediate immune–extracellular matrix and vascular-inflammatory interactions (Figure 3). GO enrichment revealed terms such as response to lipopolysaccharide, inflammatory response, response to oxidative stress, angiotensin maturation, and proteolysis, indicating that this module is strongly linked to immune activation, vascular remodeling, and oxidative imbalance. KEGG analysis identified pathways including AGE–RAGE signaling in diabetic complications, fluid shear stress and atherosclerosis, lipid and atherosclerosis, and renin–angiotensin system. Although these are often associated with cardiovascular or metabolic conditions, they share pathological mechanisms with atopic dermatitis, including chronic inflammation, barrier dysfunction, and oxidative stress.

Cluster 2 was enriched with *PPARG*, *JUN*, and *CASP3*, which are widely recognized for their influence on transcriptional regulation, inflammation, and programmed cell death. GO terms such as positive regulation of miRNA transcription, negative regulation of signaling receptor activity, collagen catabolic process, and response to hypoxia suggest that this module operates at the interface of transcriptional control, extracellular matrix remodeling, and stress-induced receptor signaling. KEGG pathways such as IL-17 signaling, TNF signaling, non-alcoholic fatty liver disease, and pathways in cancer were significantly enriched, consistent with the module’s role in amplifying inflammatory cascades, metabolic imbalance, and immune-mediated tissue injury.

Cluster 3 contained *APP* and *ALB*, genes with roles in oxidative stress regulation, protein transport, and barrier integrity. GO terms such as positive regulation of miRNA transcription, extracellular matrix disassembly, protein catabolic process, and response to hypoxia were enriched, highlighting the module’s involvement in cellular stress responses, transcriptional regulation, and matrix remodeling. In KEGG analysis, *APP* was related to the arachidonic acid metabolism, linoleic acid metabolism, PPAR signaling, and serotonergic synapse, many of which are associated with lipid mediator synthesis and inflammation resolution. Although *ALB* was not associated with any statistically significant KEGG pathways, its well-established physiological roles in osmotic pressure regulation and antioxidant defense, suggest potential indirect involvement in lipid metabolism and inflammatory modulation. Together, the three clusters represent different yet complementary aspects of AD pathology. Specifically, Cluster 1 drives barrier breakdown and inflammation, Cluster 2 amplifies immune-mediated tissue injury, and Cluster 3 contributes to metabolic regulation and inflammation resolution.

### 2.4. Transcriptional and Post-Transcriptional Regulatory Network Analysis of Top 3 Hub Genes

The top 3 hub genes, *ALB*, *CASP3*, and *PPARG*, were selected for detailed transcriptional and post-transcriptional regulatory network analysis, based on their highest rankings across multiple centrality metrics, their distinct functional clustering, and their complementary roles in AD pathology. Transcription factor (TF)–hub gene and microRNA (miRNA)–hub gene interaction networks were constructed to reveal the upstream regulatory mechanisms controlling these hub genes [24].

**Figure 4:**
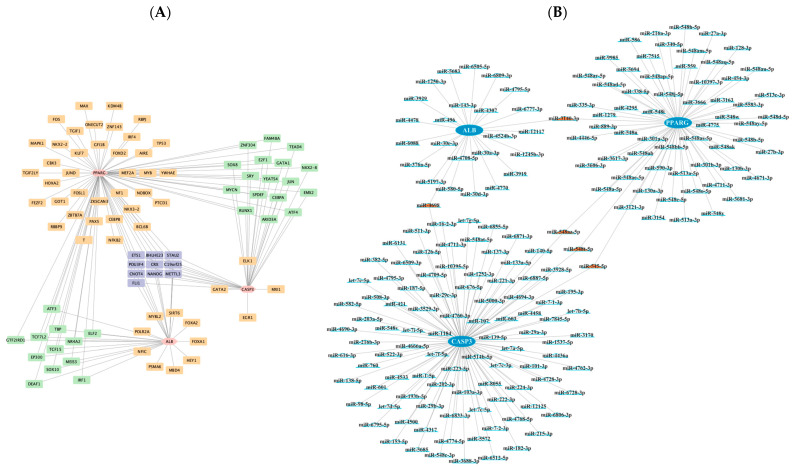
Gene regulatory interaction networks of hub genes *ALB*, *CASP3*, and *PPARG*. (**A**) Hub gene-transcription factor (TF) interaction network. Hub genes appear in pink; shared TFs (interacting with all three hub genes) are shown in purple; TFs interacting with two hub genes are green; TFs interacting with only one hub gene are shown in orange. (**B**) Hub gene-miRNA interaction network. Shared miRNAs (targeting all three hub genes) are shown in orange; unique miRNAs are shown in blue; hub genes are displayed as pink ellipses.

A total of 89 TFs were identified as potential regulators, among which 10 (e.g., *SP1*, *RELA*, *NFKB1*, *CEBPB*) exhibited concurrent interactions with all three hub genes, suggesting the presence of a shared transcriptional control axis (Figure 4A). Seventeen TFs co-regulated *CASP3* and *PPARG*, while 12 TFs were common to *ALB* and *PPARG*. Functional annotation indicated that these shared TFs are predominantly involved in inflammatory and stress-responsive transcriptional programs, notably NF-κB and AP-1 signaling. In the TF–hub gene interaction network, NF-κB–related transcription factors (e.g., *NFKB1*, *RELA*) emerged as central upstream nodes, linking inflammatory stimuli to CASP3-mediated apoptotic pathways and to PPARG-dependent lipid metabolic regulation. 

Previous studies have shown that NF-κB signaling triggers pro-apoptotic pathways, leading to caspase-3 activation and subsequent keratinocyte apoptosis, which may contribute to skin barrier disruption [25,26]. In parallel, NF-κB and PPARG interactions have been reported to influence lipid metabolic and anti-inflammatory responses in other inflammatory disorders, suggesting a potential regulatory mechanism in AD, although direct evidence remains limited [27,28].

The miRNA–hub gene interaction network (Figure 4B) revealed several post-transcriptional regulators with overlapping targets. Hsa-miR-3140-3p was predicted to target both *ALB* and *PPARG*, while hsa-miR-4698 was associated with *ALB* and *CASP3*. Three miRNAs, including hsa-miR-548az-5p, hsa-miR-5481-5p, and hsa-miR-545-5p, were found to co-regulate *CASP3* and *PPARG*. Findings from previous investigations reveal that members of the miR-548 family participate in multiple post-transcriptional processes, such as the modulation of mRNA stability, translational repression, and the feedback regulation of inflammatory signaling pathways. Additionally, some members of the miR-548 family are associated with apoptosis and lipid metabolism [29,30]. Although direct evidence for their activity in keratinocytes or atopic dermatitis is limited, their predicted targeting of *CASP3* and *PPARG* suggests a role in fine-tuning apoptosis–lipid metabolism cross-talk, as well as in context-specific regulation of barrier repair pathways. Notably, no single miRNA was predicted to regulate all three hub genes simultaneously. This finding supports the idea that transcriptional control via TFs provides a broad-spectrum regulatory framework, whereas miRNAs confer precision and specificity in post-transcriptional modulation.

### 2.5. Molecular Docking Validation of Gut Microbiota-Derived Metabolites with ALB, CASP3, and PPARG

**Figure 5:**
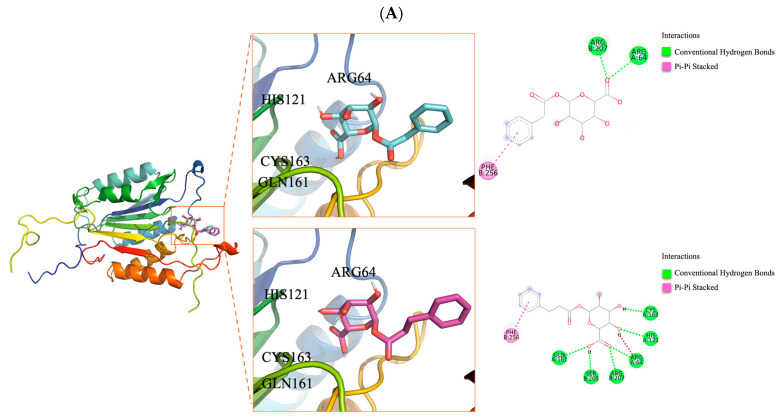

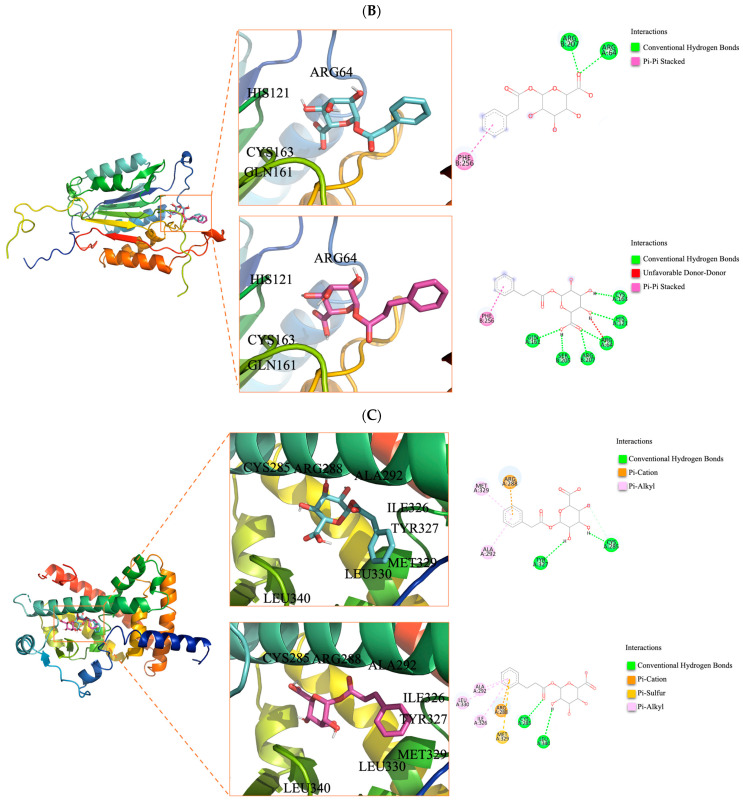
Molecular docking visualizations of THPOC and PM38 with three key target proteins. Each complex is shown with a 3D structure (left), detailed 3D view of the ligand-binding site, and 2D ligand-residue interaction map (right). Ligands are color-coded: THPOC (3,4,5-trihydroxy-6-(2-phenylacetyl)oxyoxane-2-carboxylic acid) in cyan, PM38 (propafenone metabolite 038) in magenta: (**A**) ALB, (**B**) CASP3, (**C**) PPARG. The boxes indicate the binding sites and the dashed lines show the interactions, which are color-coded according to the legend.

All 31 gut metabolites predicted from DOH-recommended medicinal plants were docked against the three top-ranked hub proteins implicated in the pathophysiology of AD (ALB, CASP3, and PPARG) to evaluate potential binding modes and affinities. Table 3 lists the top five highest-scoring metabolites for each protein target, alongside their native ligands and approved or investigational drugs used for pharmacological benchmarking. Figure 5 illustrates representative docking poses and interaction maps for two selected candidates with notable binding profiles across the targets. The complete docking dataset for all 31 screened metabolites is provided in Supplementary Table S7.

ALB showed a pronounced preference for aromatic-rich gut metabolites (Table 3). Among these, 3,4,5-trihydroxy-6-(2-phenylacetyl)oxyoxane-2-carboxylic acid (THPOC) achieved the highest docking score of −9.36 kcal/mol, surpassing the approved drug warfarin (−9.10 kcal/mol), as shown in Figure 5A. THPOC formed a distinctive π–π stacking triad at TYR138 and TYR161, supported by hydrogen bonding to ARG117. Propafenone_met038 (PM38) also showed strong affinity (−9.25 kcal/mol), forming π–π stacking interactions with TYR138, along with hydrogen bonding to TYR161 and ARG117. Aromatic substitution contributed to stabilizing interactions within the hydrophobic pocket of ALB, suggesting enhanced binding stability relative to non-aromatic analogs.

CASP3 binding profiles revealed a preference for ligands capable of forming dense hydrogen-bond networks in proximity to the catalytic residues (Table 3). As indicated in Figure 5B, PM38 yielded the strongest metabolite–protein interaction (−7.40 kcal/mol), engaging ARG207, ARG64, HIS121, CYS163, SER205, and GLN161 via hydrogen bonds, and anchoring through π–π stacking at PHE256. THPOC followed closely (−7.20 kcal/mol), forming hydrogen bonds with ARG207 and ARG64 and replicating the π–π contact at PHE256. Both metabolites matched or exceeded the docking scores of the co-crystallized ligand z-DEVD-cmk (−7.02 kcal/mol) and the investigational drug emricasan (−8.60 kcal/mol). Such interaction patterns highlight the capacity of gut-derived metabolites to mimic or surpass clinically relevant caspase inhibitors in active-site stabilization.

For PPARG, high-affinity binding was observed with metabolites combining aromatic rings and polar functional groups, enabling simultaneous hydrophobic and hydrogen-bond interactions (Table 3). PM38 achieved the top score (−7.21 kcal/mol), bridging hydrogen bonds with CYS285 and LEU340 while occupying the ligand-binding pocket through contacts with ARG288 (π-cation), MET329 (π-sulfur), and ALA292 (π-alkyl) (Figure 5C). THPOC (−7.11 kcal/mol) displayed a similar binding mode, engaging CYS285 and TYR327 through hydrogen bonding and maintaining π–cation contact with ARG288. Both metabolites showed affinities comparable to or exceeding that of the approved PPARG agonist rosiglitazone (−7.39 kcal/mol). These results indicate that gut metabolites with aromatic, drug-like scaffolds and complementary polar functionalities can achieve binding modes consistent with effective PPARG modulation. Notably, THPOC and PM38 demonstrated stronger docking affinities for ALB and PPARG than the reference drugs warfarin and rosiglitazone, respectively.

### 2.6. Dynamic and Energetic Profiling of Complexes Between Gut Metabolite and AD Target Protein

To complement molecular docking and gain deeper insight into the stability and interaction dynamics of gut metabolites with AD-associated hub proteins, MD simulations and Molecular Mechanics Poisson Boltzmann surface area (MMPBSA) binding energy analyses were performed for ALB, CASP3, and PPARG in both apo and ligand-bound forms. The top two docking hits, THPOC and PM38, were selected for their high predicted affinities and characteristic aromatic-rich binding motifs, which are hypothesized to enhance pocket stability. Each complex was simulated for 100 ns under explicit solvent conditions, and key structural (e.g., global stability, residue flexibility) and energetic parameters were evaluated to capture ligand-induced effects at the atomic scale.

#### 2.6.1. Global Stability and Structural Flexibility Using RMSD, RMSF, and Rg

Analyzing backbone deviations, residue-level fluctuations, and overall compactness helps us understand how ligand binding influences structural stability in ALB, CASP3, and PPARG. Root mean square deviation (RMSD), root mean square fluctuation (RMSF), and Radius of gyration (Rg) are key metrics used to assess these changes, revealing the impact of ligands on protein structure.

**Figure 6:**
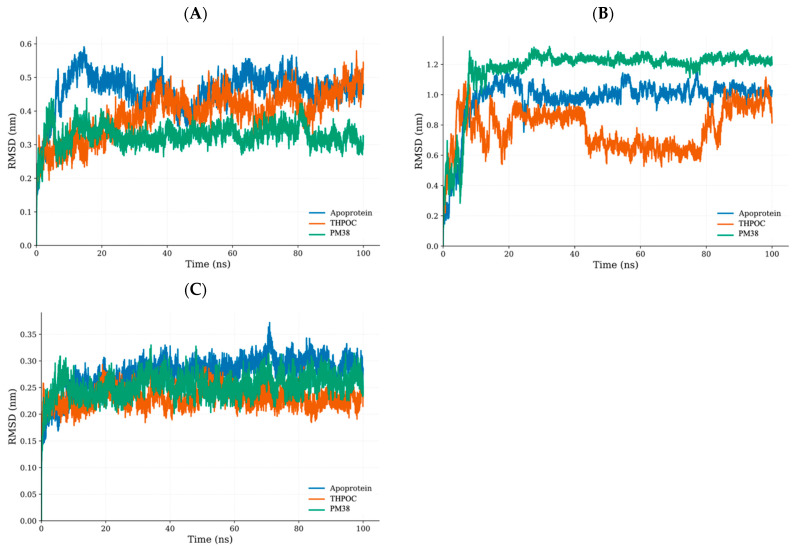
Root Mean Square Deviation (RMSD) analysis of the C-α backbone over a 100 ns simulation. RMSD trajectories comparing protein structural stability for apoprotein (blue), THPOC-bound (orange), and PM38-bound (green) complexes: (**A**) ALB, (**B**) CASP3, and (**C**) PPARG. The RMSD values reflect the dynamic behavior and conformational changes in the protein backbone throughout the simulation.

In RMSD trajectories (Figure 6), apo ALB underwent the broadest backbone deviations (~0.20–0.60 nm), which is consistent with its well-known conformational plasticity. Binding of either THPOC or PM38 narrowed this range, with the PM38 complex displaying the tightest motion (~0.25–0.55 nm), indicating pronounced conformational restraint. For CASP3, THPOC association consistently lowered RMSD relative to both the apo and PM38-bound forms, whereas PM38 slightly elevated RMSD (~1.20 nm), suggesting possible ligand-induced rearrangements. In PPARG, both THPOC and PM38 reduced RMSD from the apo baseline of ~0.28–0.36 nm to ~0.26–0.34 nm, indicating moderate stabilization without structural destabilization. This narrow shift indicates that the overall structure of PPARG remains inherently rigid, with ligand binding exerting limited yet consistent conformational tightening.

**Figure 7:**
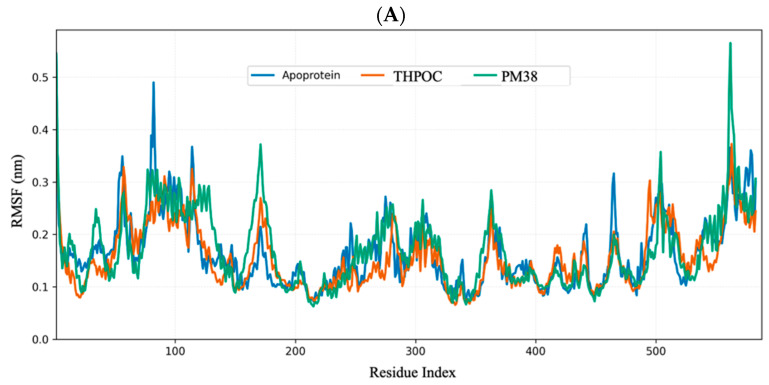

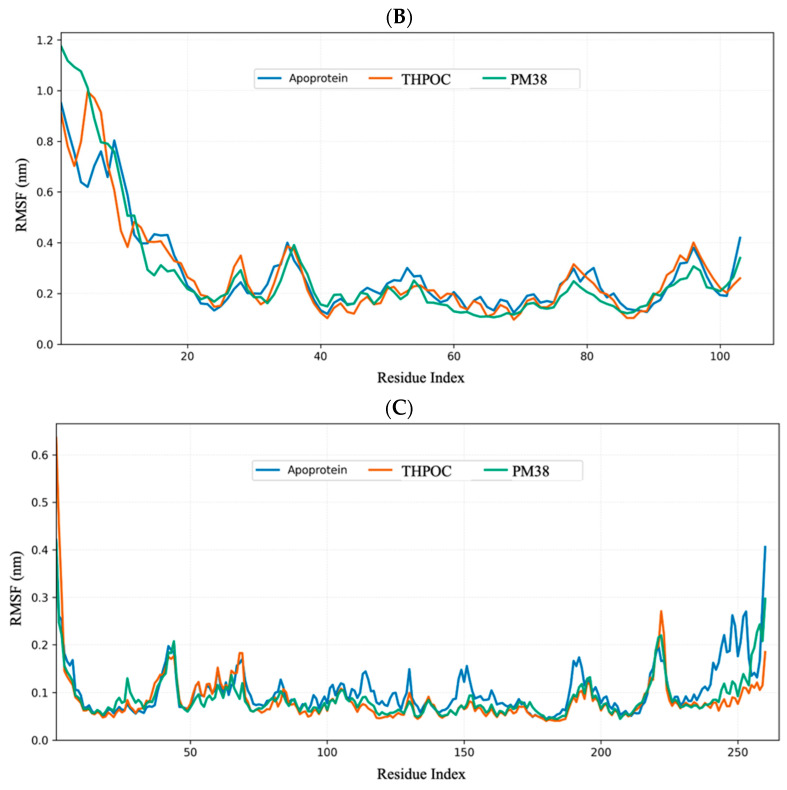
Root Mean Square Fluctuation (RMSF) of C-α atoms over residue indices during a 100 ns simulation. RMSF profiles of residues for apoprotein (blue), THPOC-bound (orange), and PM38-bound (green) forms of: (**A**) ALB, (**B**) CASP3, and (**C**) PPARG. Higher RMSF values indicate increased flexibility of specific residues, which may reflect dynamic loop regions or surface-exposed areas. Lower RMSF values in critical binding or structural regions suggest enhanced stability upon ligand binding.

As shown in Figure 7, the RMSF profiles indicated that the terminal and loop segments of ALB served as the primary mobility hotspots. Both ligands suppressed these fluctuations, particularly within the hydrophobic core. In CASP3, the flexible N-terminal region was responsible for the mobility of the apo form, but ligand binding reduced movement across multiple domains. For PPARG, the apo state showed increased flexibility in the C-terminal tail, which became significantly more rigid upon metabolite binding.

**Figure 8:**
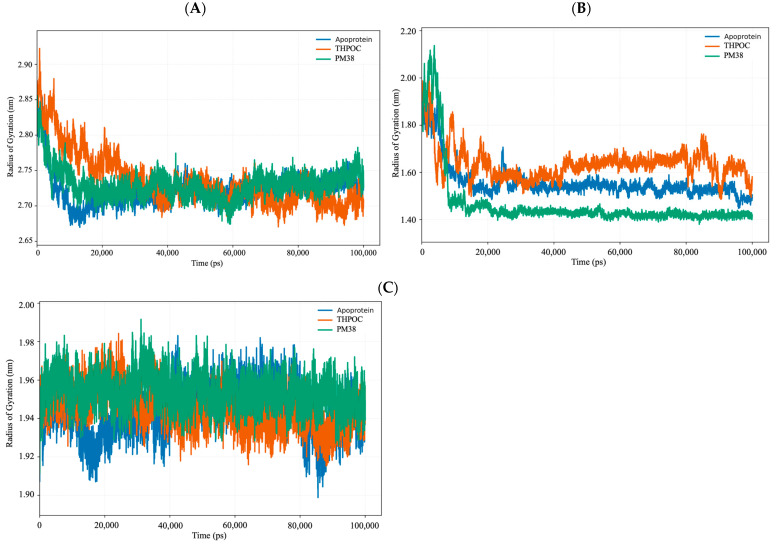
Radius of gyration (Rg) trajectories indicating compactness of protein structures over 100 ns. Rg plots comparing structural compactness and folding behavior of apoprotein (blue), THPOC-bound (orange), and PM38-bound (green) states: (**A**) ALB, (**B**) CASP3, and (**C**) PPARG. Stable and lower Rg values suggest better-maintained tertiary structure during simulation.

The Rg results (Figure 8) reflected these stability trends. Apo ALB began with a larger radius of gyration (~2.85 nm) before compacting to ~2.70–2.75 nm, while ligand-bound states maintained slightly lower and more stable values throughout the simulation. In CASP3, the apo form remained compact (~1.50–1.60 nm), THPOC binding caused mild expansion (~1.60–1.70 nm), and PM38 promoted further compaction, consistent with its stabilizing influence. PPARG systems showed minimal Rg fluctuation across apo and ligand-bound forms, indicating that its tertiary structure is inherently well-maintained and unaffected by ligand binding.

Collectively, these metrics indicate that both THPOC and PM38 reinforce structural persistence in ALB and PPARG. In CASP3, THPOC primarily attenuates conformational fluctuations, while PM38 enhances compactness, highlighting two distinct stabilization modes with potential relevance for ligand design.

#### 2.6.2. Interaction Persistence via Hydrogen Bond Dynamics

**Figure 9:**
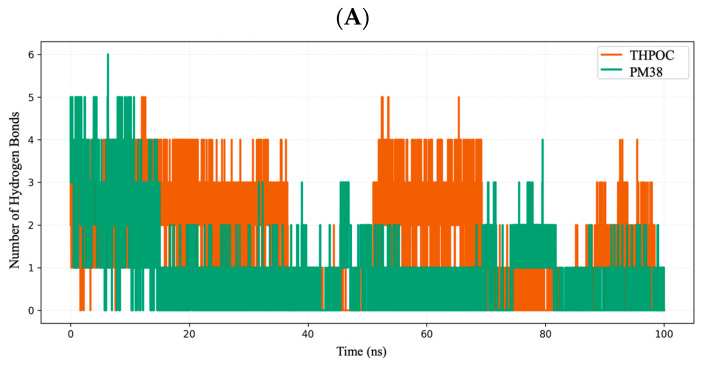

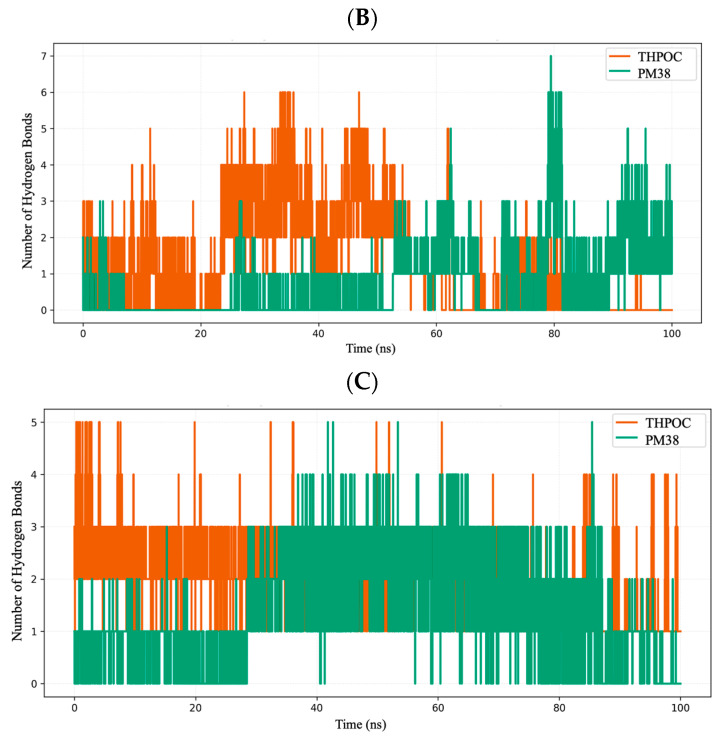
Hydrogen bond (HB) formation during 100 ns MD simulation. Time-dependent analysis of hydrogen bond formation for protein-ligand complexes: THPOC (orange) and PM38 (green), across: (**A**) ALB, (**B**) CASP3, and (**C**) PPARG. Hydrogen bond frequency reflects the stability and binding strength of the protein-ligand complexes.

Hydrogen bond analysis revealed distinct patterns of interaction persistence across ALB, CASP3, and PPARG (Figure 9). In ALB, THPOC consistently maintained hydrogen bonds, particularly during the first 60 ns, indicating sustained and stable contacts within the binding site. In contrast, PM38 exhibited more pronounced fluctuations in hydrogen bond count, suggesting intermittent binding and reduced hydrogen bond persistence. For CASP3, THPOC formed a higher number of hydrogen bonds, especially in the 20–50 ns interval, suggesting favorable stability within this time window. The PM38–CASP3 complex formed fewer and less stable hydrogen bonds during the simulation. In PPARG, the trend was reversed, with PM38 sustaining frequent and stable hydrogen bonds between 30 and 80 ns, whereas THPOC exhibited minimal hydrogen bonding beyond the first 30 ns. Overall, these observations indicate that THPOC preferentially forms more stable hydrogen bond networks with ALB and CASP3, while PM38 achieves greater interaction persistence when bound to PPARG. This observation aligns with molecular docking results, which predicted stronger affinity of THPOC for ALB and CASP3, and a preference for PM38 in PPARG, reinforcing the complementary nature of both ligands in targeting distinct AD-related proteins.

#### 2.6.3. Conformational Effects from Principal Component Analysis

**Figure 10:**
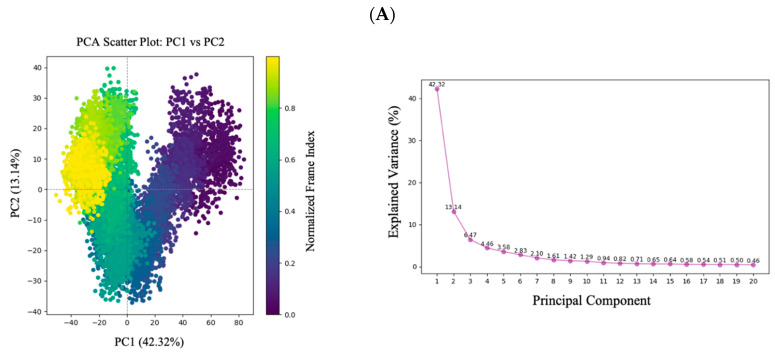

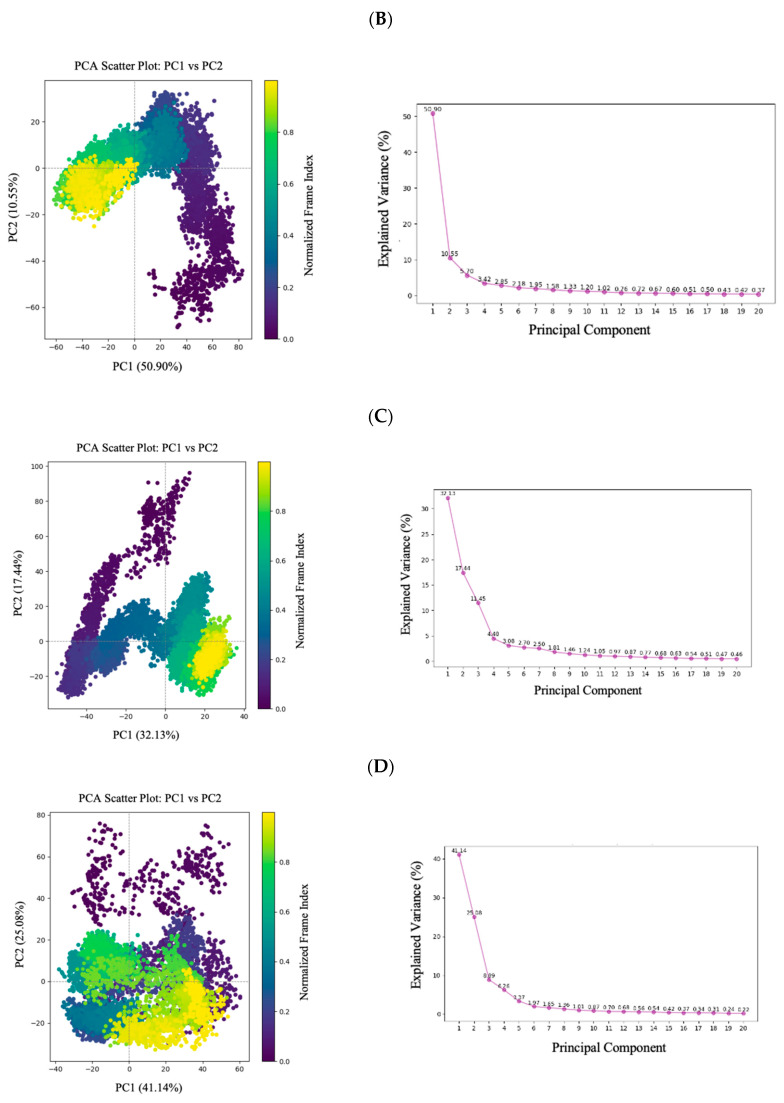

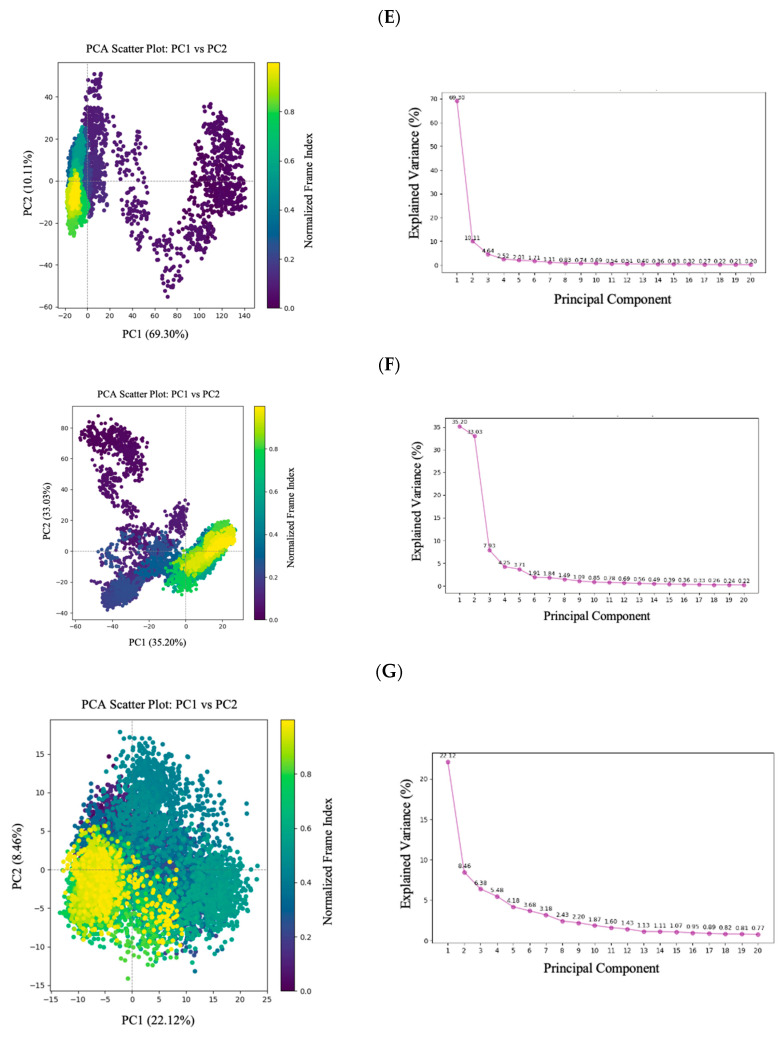

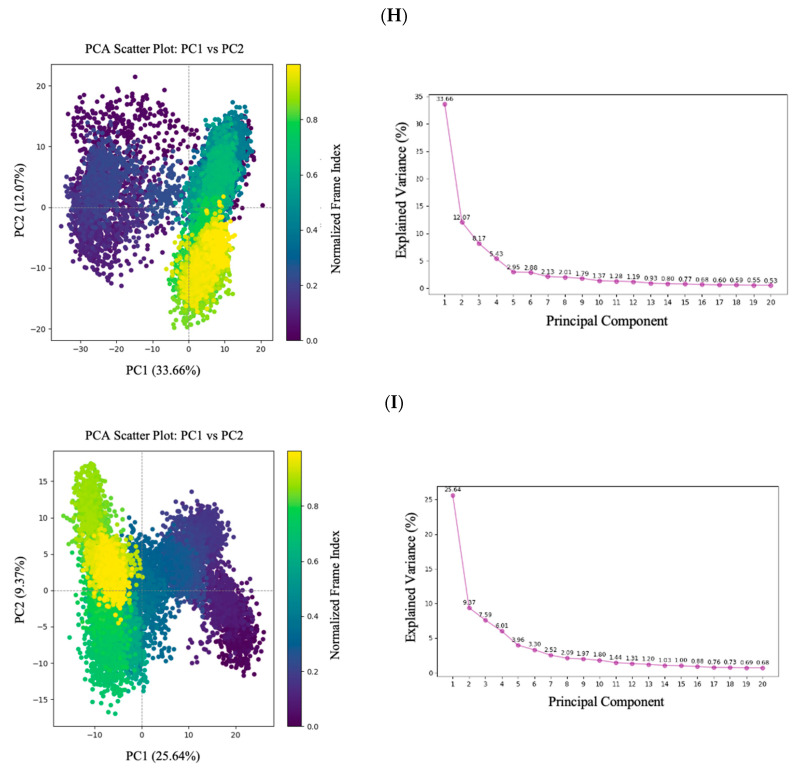
Principal Component Analysis (PCA) of protein conformational dynamics. Scatter plots and scree plots showing the dominant motions captured by PC1 and PC2 for each system across the 100 ns trajectory: (**A**) ALB-THPOC, (**B**) ALB-PM38, (**C**) ALB (apoprotein), (**D**) CASP3-THPOC, (**E**) CASP3-PM38, (**F**) CASP3 (apoprotein), (**G**) PPARG-THPOC, (**H**) PPARG-PM38, (**I**) PPARG (apoprotein). Scree plots represent the variance explained by each principal component. Dashed lines in the PCA plots indicate the zero coordinates of PC1 and PC2, serving as reference axes to visualize the distribution of conformational states.

As displayed in Figure 10, Principal component analysis (PCA) was performed to characterize large-scale conformational changes in ALB, CASP3, and PPARG upon ligand binding. In ALB, the PM38-bound system showed a broader distribution along PC1 (50.90%) and PC2 (10.55%) compared to THPOC (PC1: 42.32%, PC2: 13.14%) and the apo protein (PC1: 32.13%, PC2: 17.44%). These results suggest that PM38 induced more extensive directional motion and enhanced flexibility.

For CASP3, the PM38 complex exhibited the largest structural variance, with PC1 accounting for 69.30% and PC2 for 10.11% of the motion, accompanied by distinct clustering in the scatter plot, indicating pronounced ligand-induced rearrangements. The THPOC-bound form (PC1: 41.14%, PC2: 25.08%) and the apo form (PC1: 35.20%, PC2: 33.30%) displayed more restricted motion, reflecting conformationally constrained states.

In PPARG, PM38 binding resulted in a moderate expansion of the conformational space (PC1: 33.66%, PC2: 12.07%), while THPOC (PC1: 22.12%, PC2: 8.46%) and the apo form (PC1: 25.64%, PC2: 9.37%) maintained tighter, more compact distributions.

Overall, PCA revealed that PM38 generally promoted greater sampling of conformational space in ALB, CASP3, and PPARG, with the most substantial effect observed in CASP3. In contrast, THPOC and the apo forms were associated with more restricted conformational landscapes, consistent with their lower RMSD variability and greater structural compactness.

#### 2.6.4. Energetic Profiles from MMPBSA Calculations

The binding free energy components for each protein–ligand complex are summarized in Table 4, including van der Waals, electrostatic, polar solvation, nonpolar solvation, gas-phase, solvation, and total binding energy. These parameters provide a quantitative decomposition of the stabilizing and destabilizing forces driving ligand association, complementing the structural and dynamic analyses.

In the ALB system, THPOC exhibited strong van der Waals (−36.37 kcal/mol) and electrostatic (−19.59 kcal/mol) contributions, partially offset by polar solvation penalties (+38.18 kcal/mol), yielding a net binding energy of −23.06 kcal/mol. PM38 showed even stronger van der Waals interactions (−39.95 kcal/mol) but minimal electrostatic contribution (−3.14 kcal/mol), suggesting that its binding is predominantly hydrophobic. This lack of electrostatic stabilization is consistent with the weaker hydrogen bond persistence observed for PM38 in ALB (Figure 9A) and its broader conformational sampling in PCA (Figure 10C). Consequently, the net binding free energy of PM38 (−18.96 kcal/mol) was less favorable than THPOC (−23.06 kcal/mol). The mixed hydrophobic–electrostatic profile of THPOC appears to confer greater binding stability, aligning with its more compact and stable RMSD trajectory. Notably, docking scores also favored THPOC for ALB, consistent with the observed energetic advantage.

For CASP3, both ligands bound less tightly, with total binding energies of −10.01 kcal/mol for THPOC and −9.36 kcal/mol for PM38. While the van der Waals and electrostatic contributions were relatively balanced, the smaller magnitude of gas-phase stabilization and higher solvation penalties indicate that the CASP3 binding site is less complementary to these ligands. This observation agrees with the moderate hydrogen bond counts and weaker conformational restriction seen in RMSD and PCA analyses. Docking results also showed smaller binding score differences between the two ligands in CASP3, reflecting this reduced compatibility.

In PPARG, the energetic separation between ligands was most evident. THPOC recorded the lowest total binding energy (−33.10 kcal/mol) of all complexes, driven by highly favorable gas-phase interactions (−60.88 kcal/mol) and strong van der Waals contributions. PM38 also achieved substantial binding (−26.64 kcal/mol) through hydrophobic and gas-phase stabilization, but its larger polar solvation penalty reduced its net affinity. These trends parallel the interaction persistence results where THPOC formed stable hydrogen bonds over extended timescales and matched the docking outcome, which predicted PPARG as a high-affinity target for THPOC.

Taken together, MMPBSA analysis shows that THPOC consistently outperforms PM38 in ALB and PPARG due to synergistic hydrophobic and electrostatic stabilization, whereas CASP3 yields only modest affinities for both ligands. The agreement between energetic profiles, docking predictions, and dynamic stability metrics strengthens the view that THPOC may exert broader and more potent stabilizing effects on AD-related proteins. To further support this energetic distinction and provide a broader view of ligand–target interaction dynamics, a comparative plot summarizing hydrogen bond persistence, contact residue numbers, and binding free energies across all major complexes was presented (Figure S2). This integrated visualization underscores distinct binding preferences and supports the complementary, target-specific roles of THPOC and PM38 in stabilizing AD-relevant proteins.

### 2.7. DFT-Based Quantum Chemical Analysis of Metabolites

DFT based quantum chemical analysis was performed as the final step to characterize the electronic properties and stability of the two top-performing gut-derived metabolites, THPOC and PM38, alongside the positive control drug abrocitinib, an FDA-approved treatment for moderate-to-severe AD. These metabolites were selected based on their high affinity and consistent interaction profiles with ALB, CASP3, and PPARG. The DFT analysis aims to provide a quantum-level understanding of their molecular behavior, further supporting their potential as modulators of AD-associated protein targets.

#### 2.7.1. Structure Optimization and Energy Corrections

**Figure 11:**
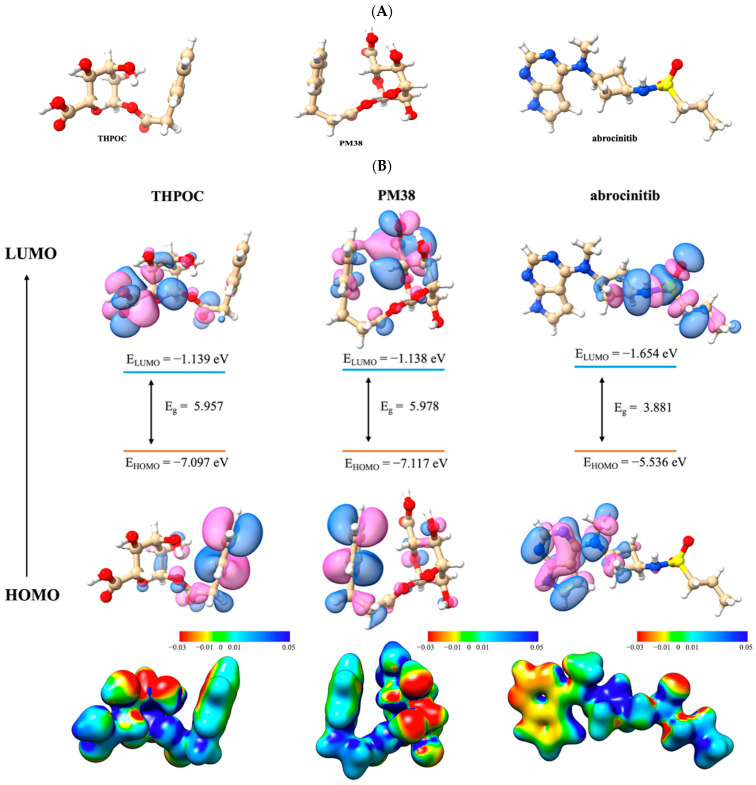

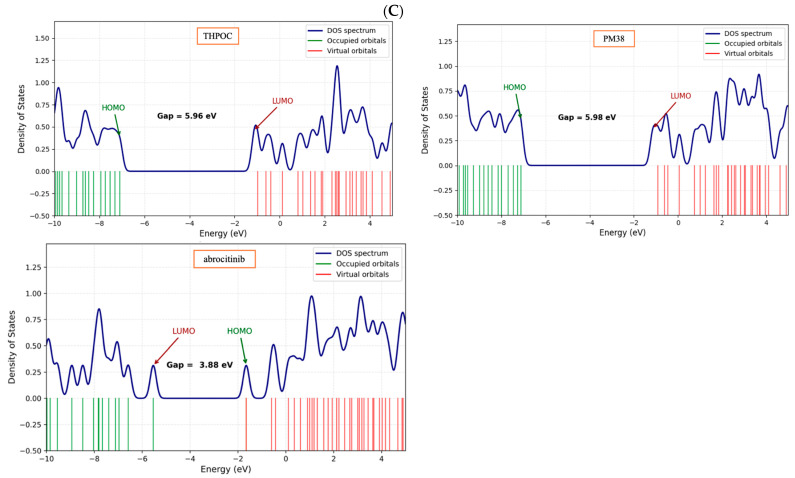
DFT-based geometric, electronic and energetic profiling of THPOC, PM38 and control drug. (**A**) Optimized geometries obtained via DFT calculations (**B**) Frontier molecular orbitals (HOMO, LUMO) and electrostatic surface potentials (ESP). Blue represents the positive orbital phase; pink represents the negative phase. Red and blue in ESP maps indicate electron-rich (negative potential) and electron-deficient (positive potential) regions, respectively. (**C**) Density of states (DOS) analysis. The blue line shows the overall DOS spectrum; green and red areas represent occupied and unoccupied orbitals, respectively. All panels correspond to THPOC, PM38, and abrocitinib.

The optimized geometries and corresponding energetic parameters of the two top-performing gut-derived metabolites, THPOC and PM38, are summarized in Figure 11A and Table 4. Optimization energies were −1144.32 Eh for THPOC and −1183.62 Eh for PM38, confirming their quantum-level structural stability under physiological simulation conditions.

Dipole moments were 3.62 Debye for THPOC and 3.75 Debye for PM38, indicating moderate polarity. This polarity profile is consistent with the balanced hydrophobic and polar interactions observed in docking and MD simulations, which may facilitate multi-target binding across ALB, CASP3, and PPARG without over-reliance on electrostatic forces. The positive control abrocitinib exhibited a more negative optimization energy (−1367.71 Eh), indicating greater intrinsic thermodynamic stability, and a substantially higher dipole moment (6.67 Debye).

The higher polarity of abrocitinib is consistent with its *JAK1* kinase inhibition mechanism, which depends on polar contacts within the ATP-binding site. While abrocitinib demonstrates high stability and strong polarity optimized for single-target inhibition, THPOC and PM38 maintain sufficient quantum stability and structural adaptability. These properties may be responsible for their ability to engage multiple AD-related proteins with stable binding profiles, as observed in preceding MD and docking results.

#### 2.7.2. Frontier Molecular Orbitals (FMO) Analysis

The frontier molecular orbital (FMO) profiles of THPOC and PM38, visualized in Figure 11B, revealed notable similarities in electronic configuration. The highest occupied molecular orbital (HOMO) energies were −7.10 eV for THPOC and −7.12 eV for PM38, while the lowest unoccupied molecular orbital (LUMO) energies were −1.14 eV. Both metabolites exhibited a wide HOMO–LUMO energy gap (5.96 eV for THPOC and 5.98 eV for PM38), indicative of high electronic stability and relatively low intrinsic chemical reactivity. Such a wide gap suggests that these molecules are less prone to spontaneous electron transfer, which is consistent with the stable ligand–protein complexes observed in MD simulations.

Electrophilicity analysis further differentiated the two metabolites. PM38 displayed the highest electrophilicity index (ω = 0.12), suggesting a stronger propensity to accept electrons and engage in electrophilic interactions, which may favor binding to electron-rich residues in target proteins such as PPARG. THPOC, with a lower ω value, demonstrated a more balanced donor–acceptor profile, aligning with its stable hydrogen bond persistence across multiple targets in MD analysis.

In contrast, the positive control abrocitinib exhibited a significantly narrower HOMO–LUMO gap (3.88 eV) compared to the metabolites, reflecting higher chemical reactivity and potentially greater electron mobility. Its electrophilicity and other global reactivity descriptors were closer to those of THPOC than to those of PM38. However, abrocitinib’s narrower gap and higher polarity suggest that it is optimized for strong, specific interactions within its canonical JAK1 binding site rather than for broad, multi-target engagement. Overall, the FMO results indicate that THPOC and PM38 possess high electronic stability, with PM38 leaning toward stronger electrophilic behavior and THPOC showing balanced donor–acceptor characteristics. These properties complement the docking and MD findings, where PM38 demonstrated favorable binding to PPARG and THPOC showed robust interactions with ALB and CASP3.

#### 2.7.3. Molecular Electrostatic Potential Mapping and Density of States Analysis

The electrostatic surface potential (ESP) and density of states (DOS) of THPOC, PM38, and the positive control were analyzed to further characterize their electronic properties (Figure 11C and Table 5). ESP maps revealed distinct spatial distributions of charge density among the three molecules. THPOC and PM38 displayed moderate electron-rich regions (red) primarily surrounding aromatic oxygen atoms and carboxyl moieties, indicating potential hydrogen bond acceptor sites. Electron-deficient regions (blue) were concentrated near aromatic ring edges, suggesting favorable electrophilic interaction sites. Abrocitinib exhibited broader and more intense electron-rich zones, reflecting its higher dipole moment (6.67 Debye) and strong polarity, which may favor polar contacts within kinase active sites. These patterns are consistent with the interaction hot spots identified in docking and MD simulations, where THPOC and PM38 achieved stable hydrogen bonding within ALB and PPARG pockets.

DOS plots showed clear separation between occupied (HOMO) and unoccupied (LUMO) molecular orbitals for all compounds. THPOC and PM38 had the same LUMO energies (−1.14 eV) and HOMO energies (−7.10 eV and −7.12 eV, respectively), resulting in wide HOMO–LUMO gaps of 5.96–5.98 eV. These features suggest high electronic stability and limited chemical reactivity, supporting sustained interactions with diverse protein targets in a physiological environment. Abrocitinib displayed a narrower gap of 3.88 eV, implying higher electron mobility and potentially greater reactivity, consistent with its ATP-competitive kinase inhibition mechanism.

Together, the ESP and DOS analyses emphasize the notion that THPOC and PM38 combine moderate polarity with electronic stability, supporting their adaptability for binding multiple AD-related targets. In contrast, the high polarity and narrower energy gap of abrocitinib are consistent with a single-target, high-affinity mode of action. These findings extend the docking and MD results by providing a quantum-level explanation for the stability and multi-target capability of the selected metabolites. These findings support the proposed systems-level hypothesis that gut microbiota-derived metabolites from medicinal plants can simultaneously modulate multiple AD-relevant targets through combined transcriptional, post-transcriptional, and structural mechanisms.

## 3. Discussion

Recent research in AD highlights its multifactorial nature, involving complex crosstalk between immune dysregulation, skin barrier dysfunction, and microbial metabolic interactions [31]. Traditional therapeutic strategies, such as topical corticosteroids, calcineurin inhibitors, biologics (e.g., dupilumab), and Janus kinase inhibitors, have significantly improved symptom control in AD. However, their long-term use is often limited by high cost, adverse effects, and variable efficacy across different AD phenotypes [32]. These immunomodulatory therapies typically target single cytokine pathways, such as IL-4/IL-13, limiting their effectiveness in heterogeneous or refractory AD subtypes. These limitations have prompted a growing interest in discovering therapeutics that can modulate multiple pathogenic pathways with safety profiles.

Two metabolites, THPOC and PM38, exhibited strong interactions with three key AD relevant hub proteins (ALB, CASP3, and PPARG). These interactions were characterized by favorable docking scores, stable binding modes, and complementary hydrogen bonding profiles. MD simulations revealed consistent structural stabilization and reduced residue fluctuation across protein–ligand complexes. MMPBSA energy analysis further confirmed favorable binding energetics, with THPOC showing stronger affinity for ALB and CASP3, and PM38 for PPARG. These results highlight the potential of THPOC and PM38 as multi-target immunometabolic modulators capable of simultaneously addressing immune signaling, apoptosis, and lipid-mediated barrier repair in AD.

To clarify the biological implications of each module, functional pathways enriched by the predicted metabolites were mapped onto corresponding hub gene clusters. A broad spectrum of AD-relevant pathways was modulated, including IL-17, TNF, AGE–RAGE, oxidative stress, and lipid metabolism. In Cluster 1, immune-fibrotic signaling cascades such as TNF and AGE–RAGE were aligned with the activities of IL2 and MMP2, suggesting a role in mitigating chronic inflammation and extracellular matrix degradation [33,34]. Cluster 2 was strongly associated with PPARG and CASP3, implicating TNF signaling and apoptosis related pathways in the regulation of inflammatory injury and immune signaling and resolution [35,36]. Cluster 3, involving ALB and APP, showed enrichment in oxidative stress response and lipid metabolic processes, such as linoleic and arachidonic acid metabolism, highlighting potential contributions to redox balance and barrier recovery [37,38].

Complementing these pathway-level insights, transcriptional and post-transcriptional regulatory analyses revealed shared upstream regulators that further consolidate the immunometabolic roles of THPOC and PM38. Hub genes such as ALB, CASP3, PPARG were commonly regulated by transcription factors including ETS1 and FLI1, known to influence inflammatory gene expression [39,40]. Moreover, miRNA analysis revealed overlapping regulators, especially the miR-548 family, implicated in lipid metabolism, apoptosis, and immune [29,30]. These regulatory elements converged with key pathways enriched by the predicted metabolites, such as IL-17, TNF, oxidative stress response, and lipid mediator biosynthesis. Among these, the PPAR signaling and linoleic/arachidonic acid metabolism pathways, both essential for epithelial barrier restoration and immune regulation, stood out. These pathways are often overlooked in conventional immunosuppressive treatments, suggesting that gut-derived metabolites could complement existing therapies by targeting underexplored mechanisms of AD pathogenesis.

The gut-skin axis provides a mechanistic bridge linking microbial metabolism with cutaneous immune responses. The biotransformation of phytochemicals into immunomodulatory metabolites presents a metabolically driven route through which gut microbiota may influence systemic immunity. Our results suggest that gut-derived metabolites regulate both canonical (e.g., cytokine suppression) and non-canonical mechanisms (e.g., PPARG-mediated transcriptional modulation and CASP3-driven apoptosis) [41,42]. Due to the known immunological heterogeneity in AD, which includes Th2-dominant, Th17/Th22-skewed, and ethnic/intrinsic subtypes, this multi-layered mode of action provides potential utility across a broader spectrum of patient profiles [43].

When compared with well-characterized natural compounds such as EGCG, glycyrrhizin, and curcumin, the gut-derived metabolites THPOC and PM38 demonstrated comparable or superior binding to core AD-relevant proteins [44]. A prominent catechin in green tea, EGCG exerts potent antioxidant activity and inhibits JAK/STAT and NF-κB signaling, thereby reducing mast cell activation and inflammatory cytokine release. Glycyrrhizin, a triterpenoid saponin from *Glycyrrhiza glabra*, has also been shown to ameliorate AD symptoms by inhibiting the pro-inflammatory mediator HMGB1, thereby reducing mast cell activation, Th2 cytokine production, and immune cell infiltration in AD mouse model [45]. Curcumin, which is isolated from Curcuma longa, reduces the expression of IL-4, IL-13, and TNF-α and restores skin barrier proteins such as filaggrin, but its clinical utility is hindered by poor bioavailability and instability due to low aqueous solubility, rapid metabolism, and pH/light sensitivity [11].

Traditional cell-based drug discovery often overlooks such metabolites, which are the actual forms circulating in vivo. By identifying stable, multi-target metabolites early in the discovery pipeline, this approach reduces reliance on trial-and-error pharmacokinetics and improves translational relevance. Recent studies have demonstrated that microbial or metabolic derivatives of natural compounds can outperform their parent forms in both bioactivity and pharmacokinetic properties. For example, tetrahydrocurcumin (THC), a gut microbiota-derived major metabolite of curcumin, has shown enhanced anti-inflammatory and antioxidant activity compared to curcumin, and was also found to inhibit the TRIP13/USP7/c-FLIP interaction in triple-negative breast cancer cells, promoting apoptotic signaling [46,47]. Regarding skin and immune regulation, baicalein, a metabolite of baicalin, has also been reported to suppress Th2 cytokines, reduce epidermal hyperplasia, and promote keratinocyte differentiation via MAPK and NF-κB modulation [48].

This perspective supports focusing the computational pipeline on gut microbiota-derived metabolites rather than parent phytochemicals. THPOC and PM38 originate from parent compounds with experimentally validated anti-inflammatory and skin protective activities. THPOC is a predicted gut metabolite of phenylacetaldehyde, a compound from *Senna alata* shown to suppress *Cutibacterium acnes*-induced inflammation in keratinocytes and monocytes by downregulating pro-inflammatory cytokines and NF-κB signaling [49]. PM38 is derived from 4-hydroxycinnamic acid, present in *Momordica charantia*, *Allium sativum*, and *Vitex negundo*, which has been demonstrated to attenuate keratinocyte activation, suppress Th1/Th2 cytokine release, and preserve tight junction proteins in AD models [50]. The established immunoregulatory and barrier-preserving effects of these parent compounds lend biological plausibility to the predicted multi-target actions of their gut-derived metabolites, strengthening the translational rationale for their further development.

In the present study, THPOC demonstrated persistent hydrogen bonding with ALB and CASP3 across 100 ns MD simulations, particularly stabilizing regions implicated in osmotic balance, antioxidant defense, and apoptosis regulation. This interaction pattern aligns with the role of ALB in maintaining barrier integrity and the regulatory function of CASP3 in keratinocyte turnover, suggesting a dual impact on skin barrier resilience and immune resolution. PM38 showed high persistence and energetic favorability in PPARG binding, engaging residues critical for lipid metabolism and anti-inflammatory transcriptional control. This is consistent with the observed enrichment of PPAR signaling and linoleic/arachidonic acid metabolism pathways, indicating potential to restore epidermal lipid composition while dampening chronic inflammation. Together, these compounds exhibit multi-target immunometabolic modulation that not only parallels but may extend beyond the mechanisms described for EGCG, glycyrrhizin, and curcumin, with the added advantage of superior pharmacokinetic compliance and structural stability under physiological conditions. The DFT-based quantum chemical characterization of THPOC and PM38 supports their mechanistic plausibility. THPOC’s mixed electrostatic-hydrophobic profile conferred stable interactions with ALB and CASP3, while PM38’s higher electrophilicity favored PPARG engagement. These profiles aligned with the MD and MMPBSA results, reinforcing the complementary nature of both compounds. Although canonical Th2 cytokines (IL-4, IL-5, IL-13) were not identified as top targets, enrichment of IL-17, TNF, and NF-κB signaling pathways points to downstream convergence with Th2 immune axes. This supports the hypothesis that gut-derived metabolites may exert their effects by modulating parallel inflammatory cascades and immune cell function.

To elucidate the functional implications of metabolite–protein interactions, target-specific hypotheses can be drawn from structural and energetic analyses. In the case of PPARG, the metabolite PM38 exhibited stable binding within the ligand-binding domain, forming hydrogen bonds with CYS285 and LEU340, and engaging ARG288 and ALA292 through π-type interactions. As CYS285 is a critical residue for agonist-induced conformational change, this interaction pattern suggests potential agonistic activity that may suppress NF-κB signaling. Despite its simpler structure, PM38 shares key pharmacophoric elements with rosiglitazone, a well-known PPARG agonist with anti-inflammatory effects, which may support its proposed mechanism of action [51,52]. For CASP3, both PM38 and THPOC interacted with the catalytic dyad residues CYS163 and HIS121, and the adjacent residue ARG207, which are essential for substrate cleavage and caspase activation. These interactions suggest a mechanism of competitive inhibition, where ligand binding could hinder substrate access to the catalytic core. Additional π–π stacking and hydrogen bonding with PHE256, a residue involved in hydrophobic pocket stabilization, may further enhance inhibitory binding affinity. Given the imbalance in cell death mechanisms in AD, these interactions may represent a novel avenue for regulating excessive keratinocyte apoptosis [53]. Regarding ALB, THPOC maintained π–π stacking with TYR138 and TYR161, and hydrogen bonding with ARG117, which are located near the protein’s major ligand-binding pocket. Albumin’s roles in lipid metabolism, oxidative stress regulation, and vascular homeostasis suggest that these binding profiles may enhance its ability to maintain immune regulation, barrier maintenance, and inflammation resolution [54].

Taken together, these mechanistic insights validate the concept that microbiota-mediated transformation can yield bioactive derivatives with optimized target specificity and improved structural stability. The observed immunometabolic modulation by THPOC and PM38 supports their potential as preventive agents for AD and illustrates the analytical value of integrating microbial biotransformation modeling with multi-layered molecular profiling in natural product research. Although the current approach allows for the comprehensive prediction of ligand–target interactions, it is still subject to the limitations of in silico models, such as inaccurate force fields, simplified solvation environments, and limited chemical diversity. These constraints may affect predictive precision but do not preclude the value of the findings in guiding experimental prioritization.

Given that these findings were derived from in silico analyses, further experimental validation will be essential to confirm their biological relevance and therapeutic applicability. Such validation may include functional assays involving barrier integrity, cytokine modulation, or keratinocyte differentiation, as well as enzyme activity assays, phosphorylation analysis, and gene expression studies using appropriate in vitro and in vivo models of AD. Collectively, this study provides a framework for translating computationally derived metabolite candidates into experimentally validated therapies, advancing the development of microbiome-informed treatments for complex inflammatory skin disorders.

## 4. Materials and Methods

### 4.1. Obtaining Chemical Compounds and Dataset Construction

Chemical constituents from ten medicinal plants approved by DOH were retrieved from the Indian Medicinal Plants, Phytochemistry, and Therapeutics 2.0 (IMPPAT 2.0; https://cb.imsc.res.in/imppat/home; accessed on 15 November 2024) [55]. The dataset was curated by removing duplicate entries to ensure integrity. In parallel, the PubChem database (https://pubchem.ncbi.nlm.nih.gov/; accessed on 15 November 2024) [56] was queried to obtain the canonical simplified molecular input line entry system (SMILES) notation for each compound, which served as input for subsequent computational analyses.

### 4.2. Biotransformational Prediction Analysis

BioTransformer 3.0 (https://biotransformer.ca/new; accessed on 28 November 2024) [57], a computational tool for simulating metabolic transformations, was employed to predict the gut microbiota-mediated conversion of phytochemicals. The human gut microbial transformation option was selected to model microbial enzymatic activity relevant to the gastrointestinal environment. This prediction provided in silico insights into the potential bioactive metabolites that could influence the gut–skin axis and thereby modulate immune-mediated skin disorders such as AD.

### 4.3. Pharmacokinetic, Drug-Likeness, and Toxicity Screening

The predicted gut microbiota-derived metabolites were evaluated for their ADMET profiles using ADMETlab 3.0 (https://admetlab3.scbdd.com/; accessed on 13 December 2024) [22], and for drug-likeness properties using SwissADME (http://www.swissadme.ch/index.php; accessed on 13 December 2024) [21]. The screening included key physicochemical descriptors such as hydrogen bond donors (≤5), acceptors (≤10), molecular weight, lipophilicity (logP ≤ 5), and topological polar surface area (TPSA ≤ 140 Å^2^), in accordance with Lipinski’s Rule of Five. Drug-likeness suitability was further examined based on a bioavailability score threshold (>0.55) and lead-likeness violations (0–1). Predicted toxicity was assessed using hERG channel inhibition risk (0–0.3), carcinogenicity (0–0.3), and hepatotoxicity (0–0.7) as filtering parameters. After applying the combined physicochemical, pharmacokinetic, and toxicity criteria, only the metabolites meeting all thresholds were retained for subsequent target prediction and network analysis.

### 4.4. Identification of Crucial Gut Microbiota-Related Targets and AD-Related Targets

Targets associated with the predicted gut metabolites were identified through two complementary computational approaches: STP (http://www.swisstargetprediction.ch/; accessed on 26 December 2024) [58] and SEA (https://sea.bkslab.org/; accessed on 26 December 2024) [59]. Target lists obtained from each platform were merged, and duplicate entries were removed to generate a non-redundant set of putative protein targets. Disease-specific gene sets linked to AD were retrieved from the DisGeNET database (https://disgenet.com/; accessed on 3 January 2025) [60] and the GeneCards database (https://www.genecards.org/; accessed on 3 January 2025) [61]. A threshold of DisGeNET ≥ 0.4 was selected based on its classification as a medium confidence level for gene–disease associations [62], while GeneCards ≥ 0.2 was used to capture genes with meaningful disease relevance based on integrated functional annotations [23]. Following the removal of redundancies, overlapping targets shared between the gut metabolite-associated proteins and AD-related genes were identified using the InteractiVenn web application (https://www.interactivenn.net/; accessed on 3 January 2025) [63], and these common targets were selected for subsequent network pharmacology analysis.

### 4.5. Construction of Protein–Protein Interaction Network, Clustering and Identification of AD-Related Hub Genes

Genes identified as common to both metabolite-related and disease-related targets were input into the STRING database (https://string-db.org/; accessed on 3 January 2025), a resource for analyzing known and predicted PPIs [64]. The PPI network was constructed using a confidence score threshold ≥ 0.4 with species restricted to Homo sapiens. This STRING threshold is commonly used to capture biologically meaningful interactions while maintaining inclusiveness in functional network construction [65]. The resulting network was visualized and analyzed using Cytoscape (v3.10.3, Cytoscape Consortium, San Diego, CA, USA) [66]. To identify biologically meaningful subnetworks, clustering was performed using the MCODE plugin, which detects densely connected modules potentially representing functional complexes within the network [67]. Hub genes were identified through topological analysis using the cytoHubba plugin [68] in Cytoscape (v3.10.3). Network connectivity was assessed using five topological parameters namely degree centrality, closeness centrality, betweenness centrality, stress, and radiality. Genes that consistently ranked highly across these multiple criteria were selected as hub genes, ensuring robustness by avoiding reliance on a single metric.

### 4.6. Gene Ontology and Pathway Enrichment Analysis

GO and KEGG pathway enrichment analysis of MCODE-derived clusters was conducted using the DAVID database (https://davidbioinformatics.nih.gov/; accessed on 7 January 2025) [69], with results categorized into Biological Process (BP), Cellular Component (CC), and Molecular Function (MF), selecting Homo sapiens as the background species. Each cluster was analyzed separately using official gene symbols as input, and the top 10 enriched GO terms and KEGG pathways were subsequently visualized using SRplot (https://www.bioinformatics.com.cn/; accessed on 7 January 2025) [70].

### 4.7. Transcription Factor and microRNA Network Creation

TFs and miRNAs regulating the identified hub genes were predicted to construct regulatory networks. Interactions between TF and hub gene were identified using the iRegulon plugin [71] in Cytoscape, which applies motif enrichment analysis to predict TF regulators. MiRNA target interactions were retrieved from miRDB (https://mirdb.org/; accessed on 13 January 2025), ensuring the inclusion of both predicted and functionally relevant regulators [72]. The integration of TFs and miRNAs enabled the construction of a regulatory network, highlighting the complex transcriptional and post-transcriptional control of hub genes. The resulting networks were visualized in Cytoscape.

### 4.8. Molecular Docking Validation

To validate target predictions from the network pharmacology analysis, molecular docking was conducted using gut microbiota-derived metabolites as ligands and three key protein targets: ALB (PDB ID: 1N5U, 1.90 Å), CASP3 (PDB ID: 2DKO, 1.06 Å), and PPARG (PDB ID: 8B94, 1.55 Å). Three-dimensional (3D) structures of ligands were retrieved from PubChem (https://pubchem.ncbi.nlm.nih.gov/; accessed on 15 February 2025) in SDF format and converted to PDB after geometry optimization, hydrogen addition, charge assignment, and removal of water molecules using UCSF Chimera (v1.18, University of California, San Francisco, CA, USA) [73].

Protein structures were downloaded from the RCSB Protein Data Bank (https://www.rcsb.org/; accessed on 26 February 2025) [74] and similarly preprocessed in UCSF Chimera. Ligand and receptor files were then converted to PDBQT format using Open Babel [75]. Co-crystallized ligands and known reference drugs were docked as controls to validate the docking protocol. Docking was performed with AutoDock Vina 1.2.0 [76] using an exhaustiveness of 8 and an energy range of 3 kcal/mol, generating 10 binding poses per ligand. Grid boxes were defined around the active sites of each protein, using a grid spacing of 0.375 Å. The grid dimensions and center coordinates were as follows:

− ALB: 14 × 14 × 14 Å (x = 31.76, y = 7.48, z = 32.64);
− CASP3: 10 × 10 × 10 Å (x = 38.02, y = 31.31, z = 26.86);
− PPARG: 14 × 14 × 14 Å (x = 13.61, y = 1.20, z = 6.11).

All simulations were executed on a macOS 15.3.1 system with Apple M1 chip and 8 GB RAM. Post-docking visualization and interaction profiling were conducted using PyMOL (v3.0, Schrödinger, LLC, New York, NY, USA) and BIOVIA Discovery Studio Visualizer (BIOVIA, San Diego, CA, USA).

### 4.9. Molecular Dynamics (MD) Simulation

To validate the molecular docking results and assess the dynamic stability of protein–ligand complexes, MD simulations were performed using GROMACS (v2023.3) [77]. The CHARMM27 force field was used for proteins, and ligand topologies were generated via SwissParam [78]. Each complex was solvated in a triclinic box with the TIP3P water model and a 1.0 nm buffer. The system was then neutralized with Na^+^ and Cl^−^ ions to achieve a physiological ionic strength of 0.1 M. Energy minimization was performed using the steepest descent algorithm for 50,000 steps. Equilibration was carried out in two phases: 100 ps of NVT at 300 K using the modified Berendsen thermostat, and 100 ps of NPT at 1 bar using the Parrinello–Rahman barostat, with position restraints applied to heavy atoms of both protein and ligand. Production runs of 100 ns were then conducted for each complex and the corresponding apo-protein under constant temperature and pressure.

Post-simulation analyses included RMSD for backbone fluctuations, RMSF for residue-level flexibility, and Rg for global structural compactness. The number and persistence of hydrogen bonds between protein and ligand were also monitored to evaluate interaction stability. These analyses provided comprehensive insight into the structural dynamics and binding behavior of the protein–ligand complexes under near-physiological conditions.

### 4.10. Principal Component Analysis and Trajectory Analysis

PCA was performed to investigate the essential motions of protein-ligand complexes during MD simulations. MDAnalysis (v2.9.0) [79] was used for computation and Matplotlib (v.3.7.3) for visualization. To remove translational and rotational artifacts, trajectories were aligned to a reference frame, focusing on the Cα atoms. The Cα coordinates from each frame were extracted and flattened into a matrix for dimensionality reduction. A covariance matrix was constructed, and PCA was applied to extract dominant motions. The results were visualized through scree plots, which displayed the proportion of variance explained by each principal component, and 2D scatter plots, which illustrated the distribution of trajectory frames along the first two components, highlighting conformational clustering and motion patterns.

### 4.11. Molecular Mechanics Poisson Boltzmann Surface Area (MMPBSA)

Binding free energies (Δ*Gbind*) were computed using the MMPBSA method to quantify protein-ligand interaction strength. Following 100 ns MD simulations, the gmx_MMPBSA tool (compatible with GROMACS 2023.3) [80] was used, along with appropriate topology and parameter files. The binding free energy was calculated as:

ΔGbind=ΔGcomplex−(ΔGprotein+ΔGligand)

where each term represents the free energy of the complex, protein, and ligand, respectively. Contributions included vacuum potential energy, polar solvation, and nonpolar solvation energies. Post-processing and analysis were conducted with gmx_MMPBSA_ana, providing thermodynamic insight into ligand binding observed in docking and MD simulations.

### 4.12. Quantum Chemistry Analysis of Metabolites Using Density Functional Theory (DFT)

DFT was employed to investigate electronic properties of metabolites that exhibited strong binding affinities to all three target proteins. Calculations were conducted using Avogadro 1.2.0 [81] for pre-processing and Orca 6.0.1 [82] for computation. Geometry optimization was carried out using the B3LYP hybrid functional and 6-311G basis set. From the optimized structures, a range of quantum chemical properties were calculated, including frontier molecular orbitals (HOMO–LUMO), vibrational frequencies, thermodynamic parameters such as zero-point energy, enthalpy, and Gibbs free energy, as well as dipole moments.

Additionally, ESP were visualized using UCSF Chimera, illustrating charge distributions relevant to molecular reactivity. The DOS was analyzed using Multiwfn (v3.8) [83] and visualized with Matplotlib, providing further insight into the electronic structure of the metabolites.

ESP represents the potential generated by a molecule’s charge distribution [84], and is defined as:

$$V\left(r\right)=\sum_{i}\frac{q_{i}}{\left|r-r_{i}\right|}-\int \frac{\rho \left(r^{\prime }\right)}{\left|r-r_{i}\right|}\;dr^{\prime }$$

$$\left\{\begin{array}{c} V(r)=\mathrm{Electrostatic}\;\mathrm{potential}\;\mathrm{at}\;\mathrm{point}\;r\;\\ q_{i}=\mathrm{Charge}\;\mathrm{of}\;\mathrm{the}\;i\mathrm{th}\;\mathrm{nucleus}\;\mathrm{positioned}\;\mathrm{at}\;r_{i}\\ \rho \left(r^{\prime }\right)=\mathrm{Electron}\;\mathrm{density}\;\mathrm{at}\;\mathrm{point}\;r \end{array}\right\}$$

ESP mapping helps identify regions prone to electrostatic interactions such as hydrogen bonding and ionic forces. Red regions indicate nucleophilic or H-bond donor sites, while blue regions denote electrophilic or H-bond acceptor sites. The DOS describes the distribution of electronic states across energy levels within a molecule and offers critical insight into its electronic configuration and chemical reactivity [85]. It can be computed using the equation below:

$$D\left(E\right)=\frac{dN}{dE}$$

$$\left\{\begin{array}{c} D(E)=\mathrm{Density}\;\mathrm{of}\;\mathrm{states}\;\mathrm{at}\;\mathrm{energy}\;E(E)\;\\ dN=\mathrm{Number}\;\mathrm{of}\;\mathrm{states}\;\mathrm{in}\;\mathrm{the}\;\mathrm{energy}\;\mathrm{range}\;[E,\;E+dE]\\ dE=\mathrm{Small}\;\mathrm{energy}\;\mathrm{interval} \end{array}\right\}$$

## 5. Conclusions

This study establishes a comprehensive computational framework that integrates gut metabolite prediction, ADMET filtering, multi-scale network pharmacology, transcriptional and post-transcriptional regulatory mapping, molecular docking, molecular dynamics, and DFT to investigate therapeutic candidates for AD. Application of this framework to ten DOH-recommended medicinal plants led to the identification of gut-derived metabolites with complementary immunometabolic activity and strong translational plausibility, supported by experimentally validated parent compounds. The study not only highlights THPOC and PM38 as promising multi-target modulators but also demonstrates the value of coupling microbial biotransformation modeling with systems-level molecular profiling, providing a preventive strategy adaptable across diverse immunological and barrier-associated profiles of AD. The integrative approach is adaptable to other complex inflammatory disorders and offers a scalable strategy for accelerating phytochemical-based drug discovery.

## Figures and Tables

**Table 1:** Physicochemical, pharmacokinetic, and toxicological profiles of ADMET-screened gut metabolites derived from 10 Philippine Department of Health (DOH)-recommended medicinal plants.

No.	Metabolites	Physicochemical ^1^					Pharmacokinetic ^1^			Toxicity ^2^		
		Molecular Weight	H-Bond Acceptor	H-Bond Donor	Topological Polar Surface Area	Lipophilicity	Lipinski Violation	Bioavailability Score	Lead-Likeness Violation	hERG	Carcinogenicity	Hepatotoxicity
			≤10	≤5	≤140	LOGP: ≤5	0: Excellent	>0.55: High	0–1: Acceptable	0–0.3: Excellent	0–0.3: Excellent	0–0.3: Excellent; 0.3–0.7: Medium
1	3,4,5-Trihydroxy-6-phenylmethoxyoxane-2-carboxylic acid (MET01)	284.26	7	4	116.45	1.02	0	0.56	0	0.04	0.09	0.33
2	3,4,5-Trihydroxy-6-(2-phenylacetyl)oxyoxane-2-carboxylic acid (THPOC)	312.27	8	4	133.52	1.08	0	0.56	0	0.02	0.06	0.31
3	3,4,5-Trihydroxy-6-propanoyloxyoxane-2-carboxylic acid (MET02)	250.20	8	4	133.52	0.14	0	0.56	0	0.01	0.20	0.41
4	6-Acetyloxy-3,4,5-trihydroxyoxane-2-carboxylic acid (MET03)	236.18	8	4	133.52	0	0	0.56	1	0.01	0.19	0.43
5	3,4,5-Trihydroxy-6-[(1,7,7-trimethyl-2-bicyclo[2.2.1]heptanyl)oxy]oxane-2-carboxylic acid (MET04)	330.37	7	4	116.45	1.15	0	0.56	0	0.03	0.15	0.39
6	3,4,5-Trihydroxy-6-[(3-methylbutanoyl)oxy]oxane-2-carboxylic acid (MET05)	278.26	8	4	133.52	0.80	0	0.56	0	0.02	0.26	0.48
7	CURCUMIN_met045 (MET06)	326.30	8	4	125.68	1.48	0	0.56	0	0.03	0.20	0.45
8	3,4,5-Trihydroxy-6-(2-hydroxyphenoxy)oxane-2-carboxylic acid (MET07)	286.23	8	5	136.68	0.20	0	0.56	0	0.02	0.15	0.43
9	6-(4-Ethenylphenoxy)-3,4,5-trihydroxyoxane-2-carboxylic acid (MET08)	296.27	7	4	116.45	0.60	0	0.56	0	0.03	0.16	0.41
10	3-Phenylpropionic acid (MET09)	150.17	2	1	37.30	1.50	0	0.85	1	0.05	0.23	0.40
11	Propafenone_met038 (PM38)	326.30	8	4	133.52	1.09	0	0.56	0	0.04	0.18	0.57
12	3,4,5-Trihydroxy-6-pentoxyoxane-2-carboxylic acid (MET10)	264.27	7	4	116.45	1.41	0	0.56	0	0.04	0.12	0.40
13	3,4,5-Trihydroxy-6-(5-methyl-2-propan-2-ylcyclohexyl)oxyoxane-2-carboxylic acid (MET11)	332.39	7	4	116.45	1.93	0	0.56	0	0.03	0.14	0.53
14	2-Ethylhexanoic acid (MET12)	144.21	2	1	37.30	1.93	0	0.85	1	0.05	0.30	0.50
15	NoName_1282 (MET13)	320.34	8	4	133.52	1.88	0	0.56	1	0.03	0.17	0.48
16	3,4,5-Trihydroxy-6-(5-methyl-2-propan-2-ylphenoxy)oxane-2-carboxylic acid (MET14)	326.34	7	4	116.45	1.67	0	0.56	0	0.03	0.20	0.54
17	6-(Decanoyloxy)-3,4,5-trihydroxytetrahydro-2H-pyran-2-carboxylic acid (MET15)	348.39	8	4	133.52	2.26	0	0.56	1	0.05	0.15	0.42
18	3,4,5-Trihydroxy-6-octanoyloxyoxane-2-carboxylic acid (MET16)	320.34	8	4	133.52	1.29	0	0.56	1	0.03	0.17	0.43
19	3,4,5-Trihydroxy-6-(2-phenylethoxy)oxane-2-carboxylic acid (MET17)	298.29	7	4	116.45	1.85	0	0.56	0	0.05	0.11	0.53
20	6-Ethoxy-3,4,5-trihydroxyoxane-2-carboxylic acid (MET18)	222.19	7	4	116.45	0.56	0	0.56	1	0.02	0.13	0.32
21	3,4,5-Trihydroxy-6-pentan-2-yloxyoxane-2-carboxylic acid (MET19)	264.27	7	4	116.45	1.37	0	0.56	0	0.02	0.21	0.45
22	3,4,5-Trihydroxy-6-(2-methylpropanoyloxy)oxane-2-carboxylic acid (MET20)	264.23	8	4	133.52	1.01	0	0.56	0	0.01	0.15	0.47
23	6-Butan-2-yloxy-3,4,5-trihydroxyoxane-2-carboxylic acid (MET21)	250.25	7	4	116.45	1.83	0	0.56	0	0.02	0.16	0.40
24	5-(3,5-Dihydroxyphenyl)-4-hydroxyvaleric acid (MET22)	226.23	5	4	97.99	0.92	0	0.56	1	0.07	0.25	0.46
25	3,4,5-Trihydroxy-6-(2-methylpropoxy)oxane-2-carboxylic acid (MET23)	250.25	7	4	116.45	1.51	0	0.56	0	0.03	0.18	0.44
26	6-(2-Butoxyethoxy)-3,4,5-trihydroxyoxane-2-carboxylic acid (MET24)	294.30	8	4	125.68	1.36	0	0.56	1	0.04	0.26	0.29
27	4-Hydroxy-5-(3-hydroxyphenyl)pentanoic acid (MET25)	210.23	4	3	77.76	1.32	0	0.56	1	0.06	0.22	0.45
28	gamma-4-Dihydroxybenzenepentanoic acid (MET26)	210.23	4	3	77.76	1.04	0	0.56	1	0.06	0.22	0.45
29	4-Hydroxy-5-(4-hydroxy-3-methoxyphenyl)pentanoic acid (MET27)	240.25	5	3	86.99	1.67	0	0.56	1	0.05	0.24	0.43
30	3,4,5-Trihydroxy-6-[(2-methylpropan-2-yl)oxy]oxane-2-carboxylic acid (MET28)	250.25	7	4	116.45	0.91	0	0.56	0	0.02	0.18	0.36
31	(2S,3S,4S,5R)-3,4,5-trihydroxy-6-[[(1S,5S)-2-methyl-3-oxo-5-propan-2-yl-2-bicyclo [3.1.0] hexanyl]oxy]oxane-2-carboxylic acid (MET29)	330.37	7	4	116.45	1.47	0	0.56	0	0.04	0.17	0.48

^1^ Data from SwissADME [21]; ^2^ Data from ADMETlab [22].

**Table 2:** The identified top 10 hub genes and their topological scores.

Genes	Name	Cytohubba Based Topological Scores				
		Degree	Betweenness	Closeness	Stress	Radiality
*ALB*	albumin	103	6084.58	147.00	47,462	4.55
*CASP3*	caspase 3	74	2173.82	131.66	21,790	4.39
*PPARG*	peroxisome proliferator activated receptor	74	1848.35	132.16	22,710	4.37
*MMP9*	matrix metallopeptidase 9	67	1290.18	127.33	14,526	4.31
*CXCL8*	C-X-C motif chemokine ligand 8	64	1971.20	125.83	19,292	4.29
*JUN*	Jun proto-oncogene	60	939.90	123.83	11,722	4.27
*IL2*	interleukin 2	48	1178.01	117.66	11,872	4.20
*ACE*	angiotensin I converting enzyme	47	651.16	116.00	8856	4.16
*APP*	amyloid beta precursor protein	46	890.12	116.33	10,308	4.18
*MMP2*	matrix metallopeptidase 2	46	354.18	115.16	5206	4.15

**Table 3:** Docking interactions of gut metabolites from Philippine DOH-recommended medicinal plants with top hub proteins.

Targets	Metabolite/Ligand	Structure	Type	Binding Affinity (kcal/mol)	Residues with H-Bond	Hydrophobic Interaction (Type)
ALB	3,4,5-Trihydroxy-6-(5-methyl-2-propan-2-ylphenoxy)oxane-2-carboxylic acid	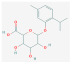	Candidate gut metabolite from DOH plants	−9.36	TYR161, ARG117	TYR138 (π-sigma), TYR161 (π-π stacked, alkyl), ILE142 (alkyl, π-alkyl)
	Propafenone_met038 (PM38)	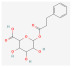		−9.25	TYR161, ARG117	TYR138 (π-π stacked)
	3,4,5-Trihydroxy-6-(2-phenylacetyl)oxyoxane-2-carboxylic acid (THPOC)	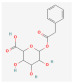		−9.01	ARG117	TYR138 (π-π stacked)
	3,4,5-Trihydroxy-6-phenylmethoxyoxane-2-carboxylic acid	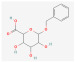		−8.79	TYR161	TYR138 (π-π stacked)
	3,4,5-Trihydroxy-6-(2-phenylethoxy)oxane-2-carboxylic acid	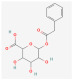		−8.69	-	TYR161, TYR138 (π-π stacked)
	HEME	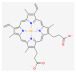	Native ligand	−13.30	LYS190	ILE142 (π-sigma), TYR161 (π-π stacked, π-alkyl), TYR138 (π-π stacked, π-alkyl, alkyl), ALA158 (π-alkyl, alkyl), ARG186, LEU154, LEU139, MET123, PHE165, PRO118 (alkyl), PHE149, ARG117, PHE134, LEU135 (π-alkyl)
	Warfarin	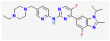	Drug	−9.10	ARG117	TYR138, PHE165 (π-π stacked), ARG117, ARG186, MET123 (π-alkyl)
CASP3	Propafenone_met038 (PM38)	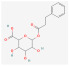	Candidate gut metabolite from DOH plants	−7.40	ARG207, ARG64, HIS121, CYS163, SER205, GLN161	PHE256 (π-π stacked)
	3,4,5-Trihydroxy-6-(2-phenylethoxy)oxane-2-carboxylic acid (THPOC)	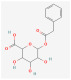		−7.30	ARG207, ARG64, HIS121, CYS163	PHE256 (π-π stacked)
	3,4,5-Trihydroxy-6-(2-phenylacetyl)oxyoxane-2-carboxylic acid	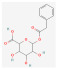		−7.20	ARG207, ARG64	PHE256 (π-π stacked)
	3,4,5-Trihydroxy-6-(2-hydroxyphenoxy)oxane-2-carboxylic acid	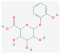		−6.93	ARG207, ARG64, HIS121, GLY122, SER205	HIS121 (π-cation), CYS163 (π-sulfur)
	3,4,5-Trihydroxy-6-(5-methyl-2-propan-2-ylphenoxy)oxane-2-carboxylic acid	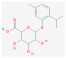		−6.88	PHE250	PHE256 (π-sigma), TRP206 (alkyl), ARG207 (π-alkyl)
	z-DEVD-cmk	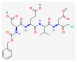	Native ligand	−7.02	LYS210, ASN208, SER209, HIS121, ARG207	PHE256 (π-π stacked)
	Emricasan	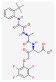	Drug	−8.60	ARG64, HIS121, SER205, GLN161	HIS121 (π-cation), CYS163 (π-sulfur), TRP206 (π-sigma), PHE256 (π-π stacked, π-alkyl)
PPARG	Propafenone_met038 (PM38)	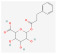	Candidate gut metabolite from DOH plants	−7.21	CYS285, LEU340	ARG288 (π-cation), MET329 (π-sulfur), ALA292, LEU330, ILE326 (π-alkyl)
	3,4,5-Trihydroxy-6-(2-hydroxyphenoxy)oxane-2-carboxylic acid (THPOC)	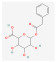		−7.11	TYR327, CYS285	ARG288 (π-cation), ALA292 (π-alkyl)
	3,4,5-Trihydroxy-6-phenylmethoxyoxane-2-carboxylic acid	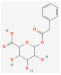		−6.99	-	ARG288 (π-cation), ALA292 (π-sigma), MET329 (amide-π stacked), ILE326 (π-alkyl)
	4-Hydroxy-5-(4-hydroxy-3-methoxyphenyl) pentanoic acid	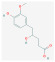		−6.86	ARG288, CYS285	CYS285 (π-sulfur, π-alkyl), LEU330 (π-alkyl, alkyl), MET364, ALA292, ARG288, ILE326 (alkyl)
	5-(3,5-Dihydroxyphenyl)-4-hydroxyvaleric acid	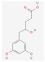		−6.83	TYR327	ARG288 (π-cation), MET329 (amide-π stacked), ALA292, ILE326 (π-alkyl)
	Q50	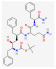	Native ligand	−8.50	GLU378	HIS425 (π-cation, alkyl), ARG234 (π-anion, alkyl), ALA233, LYS230 (alkyl), LEU377 (π-alkyl)
	Rosiglitazone	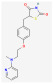	Drug	−7.39	CYS285	HIS425 (π-cation), ILE326 (π-sigma), CYS285, ARG288, LEU330 (π-alkyl)

**Table 4:** MMPBSA decomposition of binding free energies for ALB, CASP3, and PPARG complexes with THPOC and PM38.

Complex	ΔVDWAALS ^1^	ΔEEL ^2^	ΔEGB ^3^	ΔESURF ^4^	ΔGGAS ^5^	ΔGSOLV ^6^	ΔTOTAL ^7^
ALB-THPOC	−36.37	−19.59	38.18	−5.29	−55.96	32.89	−23.06
ALB-PM38	−39.95	−3.14	29.94	−5.81	−43.09	24.13	−18.96
CASP3-THPOC	−11.63	−15.72	19.36	−2.01	−27.35	17.35	−10.01
CASP3-PM38	−13.54	−12.14	18.54	−2.22	−25.68	16.32	−9.36
PPARG-THPOC	−36.01	−24.88	33.46	−5.67	−60.88	27.79	−33.1
PPARG-PM38	−39.98	−12.70	30.63	−5.59	−51.69	25.05	−26.64

THPOC: 3,4,5-Trihydroxy-6-(2-phenylacetyl)oxyoxane-2-carboxylic acid; PM38: Propafenone_ met038; Values are presented as average binding energy (kcal/mol). ^1^ ΔVDWAALS: van der Waals energy; ^2^ ΔEEL: electrostatic energy; ^3^ ΔEGB: polar solvation energy calculated using the Generalized Born model; ^4^ ΔESURF: nonpolar solvation energy; ^5^ ΔGGAS: sum of van der Waals and electrostatic energies; ^6^ ΔGSOLV: sum of polar and nonpolar solvation energies; ^7^ ΔTOTAL: total binding free energy (ΔGGAS + ΔGSOLV).

**Table 5:** Density functional theory (DFT)-derived molecular energetics, frontier orbital parameters, and reactivity descriptors of THPOC and PM38.

Compound	Molecular Energetics					Frontier Orbital Parameters				Reactivity Descriptors				
	EE+ Zero Point Energy (Eh)	EE + Thermal Energy (Eh)	Total Enthalpy (Eh)	Gibbs Free Energy (Eh)	Optimization Energy (Eh)	Dipole Moment (Debye)	HOMO (H) (eV)	LUMO (L) (eV)	Gap (L − H) (eV)	Hardness (ƞ, eV)	Softness (S, eV^−1^)	Electro-negativity (χ, eV)	Mean Energy (μ, eV)	Electro-philicity index (ω, eV)
THPOC	−1144.03	−1144.01	−1144.01	−1144.08	−1144.33	3.62	−7.10	−1.14	5.96	2.98	0.17	0.15	−0.15	0.10
PM38	−1183.30	−1183.60	−1183.27	−1183.34	−1183.62	3.75	−7.12	−1.14	5.98	2.99	0.17	0.13	−0.13	0.12
Control drug	−1367.36	−1367.69	−1367.34	−1367.41	−1367.71	6.67	−5.54	−1.65	3.88	1.94	0.26	0.15	−0.15	0.10

THPOC (PubChem ID: 131839498); PM38 (PubChem ID: 131831704); control drug (PubChem ID: 78323835) as a positive control; EE: Electronic energy; HOMO: Highest occupied molecular orbital; LUMO: Lowest unoccupied molecular orbital.

## Data Availability

Data are contained within the article or Supplementary Materials.

## Supplementary Materials

The following supporting information can be downloaded at: https://www.mdpi.com/article/10.3390/ijms262110731/s1.

## Abbreviations

The following abbreviations are used in this manuscript:

AD	Atopic dermatitis
DOH	Department of Health
DFT	Density Functional Theory
SCFAs	short chain fatty acids
EGCG	epigallocatechin gallate
MD	Molecular dynamics
STP	Swiss Target Prediction
SEA	Similarity ensemble approach
PPI	Protein–protein interaction
MCODE	Molecular Complex Detection
GO	Gene ontology
KEGG	Kyoto encyclopedia of genes and genomes
TF	Transcription factor
miRNA	microRNA
THPOC	3,4,5-trihydroxy-6-(2-phenylacetyl) oxyoxane-2-carboxylic acid
PM38	Propafenone_met038
MMPBSA	Molecular Mechanics Poisson Boltzmann surface area
RMSD	Root mean square deviation
RMSF	Root mean square fluctuation
Rg	Radius of gyration
PCA	Principal component analysis
FMO	Frontier molecular orbital
HOMO	Highest occupied molecular orbital
LUMO	Lowest unoccupied molecular orbital
ESP	Electrostatic surface potential
DOS	Density of states
